# Low-dose pro-resolving mediators temporally reset the resolution response to microbial inflammation

**DOI:** 10.1186/s10020-024-00877-w

**Published:** 2024-09-18

**Authors:** Charles N. Serhan, Nan Chiang, Robert Nshimiyimana

**Affiliations:** Department of Anesthesiology, Perioperative and Pain Medicine, Center for Experimental Therapeutics and Reperfusion Injury, Mass General Brigham and Harvard Medical School, 60 Fenwood Rd., Hale Building for Transformative Medicine 3-016, Boston, MA 02115 USA

**Keywords:** Macrophage, Neutrophil, Resolvins, Protectins, Maresins, LC–MS–MS, SPMs

## Abstract

**Background:**

Specialized pro-resolving mediators (SPMs) promote resolution of inflammation, clear infections and stimulate tissue regeneration. These include resolvins, protectins, and maresins. During self-resolving acute inflammation, SPMs are produced and have key functions activating endogenous resolution response for returning to homeostasis. Herein, we addressed whether infections initiated with ongoing inflammation alter resolution programs, and if low-dose repetitive SPM regimen re-programs the resolution response.

**Methods:**

Inflammation was initiated with zymosan (1 mg/mouse) followed by *E. coli* (10^5^ CFU/mouse) infections carried out in murine peritonitis, and exudates collected at 4-72 h. Leukocytes were enumerated using light microscopy, percentages of PMN, monocytes and macrophages were determined using flow cytometry, and resolution indices calculated. Lipid mediators and SPM profiles were established using mass spectrometry-based metabololipidomics. Repetitive dosing with a SPM panel consisting of RvD1, RvD2, RvD5, MaR1 and RvE2 (0.1 ng/mouse each, *i.p*.) was given to mice, followed by zymosan challenge. Leukocyte composition, resolution indices and RNA-sequencing were carried out for the repetitive SPM treatments.

**Results:**

*E. coli* infections initiated acute inflammation-resolution programs with temporal SPM production in the infectious exudates. Zymosan-induced inflammation prior to *E. coli* peritonitis shifted exudate resolution indices and delayed *E. coli* clearance. Lipid mediator metabololipidomics demonstrated that *E. coli* infection with ongoing zymosan-induced inflammation shifted the time course of exudate SPMs, activating a SPM cluster that included RvD1, RvD5 and MaR1 during the initiation phase of infectious inflammation (0-4 h); RvD5 and MaR1 were present also in the resolution phase (24-48 h). To emulate daily SPM regimens used in humans, a repetitive subthreshold dosing of the SPM panel RvD1, RvD2, RvD5, MaR1 and RvE2 each at 0.1 ng per mouse was administered. This low-dose SPM regimen accelerated exudate PMN clearance following zymosan-induced inflammation, and shortened the resolution interval by > 70%. These low-dose SPMs regulated genes and pathways related to immune response, chemokine clearance and tissue repair, as demonstrated by using RNA-sequencing.

**Conclusions:**

Infections encountered during ongoing inflammation in mice reset the resolution mechanisms of inflammation via SPM clusters. Low-dose SPMs activate innate immune responses and pathways towards the resolution response that can be reprogrammed.

**Supplementary Information:**

The online version contains supplementary material available at 10.1186/s10020-024-00877-w.

## Background

In the evolution of the innate immune system, the acute inflammatory response initiates the recruitment of leukocytes from circulation to defend and wall off microbial invaders (Majno and Joris 2004). Overall, this is a protective response. The summoning of leukocytes into tissue requires chemotactic signals, as all cells need gradients of chemoattractants to move (Majno and Joris 2004). In the acute inflammatory response, the influx of neutrophils is initiated by chemokines and cytokines. The lion’s share is summoned by leukotriene B_4_, a well-known pro-inflammatory chemoattractant (Malawista et al. 2008; Hopke et al. 2022; Lammermann et al. 2013). This initial phase also produces prostaglandin E_2_ that is required to enable neutrophil diapedesis, a pro-inflammatory response, by acting on endothelial cells, stimulating the induction of resolution phase mediators (Serhan 2007). The resolution phase of a normal healthy acute inflammatory response disassembles the defense battleline of neutrophils and returns the tissue to homeostasis by pro-resolving processes such as efferocytosis of apoptotic neutrophils by reparative macrophages (Serhan 2007).

In this resolution phase, omega-3 essential fatty acids eicosapentaenoic acid (EPA) and docosahexaenoic acid (DHA) are mobilized to produce potent resolvins, protectins and maresins we collectively termed the specialized pro-resolving mediators (SPMs), that each limit further neutrophil recruitment, activate efferocytosis and reduce pain (Serhan 2007; Fredman and Serhan 2024). Since the loss of neutrophils from tissues defined the resolution phase (Majno and Joris 2004; Serhan 2007), earlier we introduced quantitative resolution indices to help pinpoint the precise cellular and molecular actions of each SPM within the resolution phase (Schwab et al. 2007). These indices take into account the numbers of neutrophils in the exudate tissue, the magnitude and duration of the resolution response in vivo. See the definitions of Resolution Indices vide infra in Methods. These are useful in defining resolution agonists to control inflammation rather than inhibitors of the chemical mediators in inflammation (Schwab et al. 2007). These resolution indices are in wide use and proved useful in diverse animal models (Recchiuti et al. 2014; Korner et al. 2018; Bhattacharya et al. 2023). For example, the oral administration of RvD1 impacts peritonitis, shortening the resolution intervals (Recchiuti et al. 2014). Oral omega-3 supplementation increases SPMs and survival in murine sepsis and in peritonitis shortened the resolution interval (Korner et al. 2018).

To further address mechanisms in resolution of inflammation, in our NIH/NIGMS Program Project (P01GM095467 to C.N.S), we prepared synthetic standards for each SPM uncovered and functionally characterized by total organic synthesis to confirm potent pro-resolving functions, and deuterium-labeled SPM standards were also prepared to enable SPM identification and quantification (Hong et al. 2007). Many of these novel molecules are now commercially available, permitting confirmation of their potent pro-resolving functions by others, for example, RvD2 in reducing coronary atherosclerosis (Bardin et al. 2022), diminishing aging (Fitzgerald et al. 2023), in stimulating tissue regeneration (Dort et al. 2021), RvD1 in activating myeloid and muscle stem cells (Markworth et al. 2020), resolvins in reducing lung inflammation (Sekheri et al. 2020), as well as enabling documentation of SPM biosynthesis in humans (Blaauw et al. 2024; Barden et al. 2024; Lau et al. 2023; Hartling et al. 2021) and in animal models (Korner et al. 2019). These were also useful in documenting SPMs in severe SARS-CoV-2 infection in humans (Schwarz et al. 2021; Palmas et al. 2021), giving rise to the notion that dysregulated SPMs and lipid mediators impact the magnitude and duration of the infection (Palmas et al. 2021; Serhan et al. 2022).

In view of these and the potential threat of another global microbial challenge (Huang et al. 2024; Morse et al. 2012), we sought evidence for the production and actions of SPMs during bacterial infection in mice with ongoing inflammation. Here we report that SPM production can reset and upregulate the resolution response of inflammation.

## Materials and methods

### Murine peritonitis

Animal experimental procedures were approved by the Institutional Animal Care and Use Committee (IACUC) of Brigham and Women's Hospital (protocol no. 2016N000145). Mice were anesthetized with isoflurane, and experiments carried out with male C57BL/6 mice (6 to 8 weeks; Jackson Laboratory). Individual mice were used for each timepoint. Mice were sacrificed at designated timepoint using overdose of isoflurane following with cervical dislocation according to approved procedures by IACUC.

*E. coli and Zymosan peritonitis:* Mice were inoculated with *E. coli* (serotype O6:K2:H1; 1 × 10^5^ CFU, *i.p*.) to initiate infectious peritonitis, and exudates collected at indicated time points. Intact leukocytes were identified using trypan blue exclusion, and enumerated using a light microscope. Leukocyte subtypes were assessed using flow cytometry with anti-mouse PerCP/Cy5.5 CD45 (clone 30-F11 Biolegend, CA), anti-mouse PE-CD11b (clone M1/70, eBioscience), anti-mouse APC F4/80 (clone BM8, eBioscience), anti-mouse FITC Ly6C (clone HK1.4, Biolegend) and anti-mouse APCcy7-Ly6G (clone 1A8, Biolegend). Peritoneal exudate populations were determined as PMN (CD45 + CD11b + Ly6G + Ly6C-), monocytes (CD45 + CD11b + Ly6G-Ly6C +) or MΦ (CD45 + CD11b + F4/80 +). In select experiments, Zymosan (1 mg/mL, i.p.) was given two hours before *E. coli* inoculation. Exudate *E. coli* titers were determined by plating serial dilutions of exudates onto LB agar plates for 24 h. The *E. coli* titers were determined by counting *E. coli* colony forming units (CFU) on the plates.

*Zymosan peritonitis with repetitive SPM dosing*: *For repetitive dosing*, mice were given a SPM panel (RvD1, RvD2, RvD5, MaR1 and RvE2, 0.1 ng each/mouse, i.p.) on days 0, 2, 5, 7 and 9. Next, on day 12, the SPM panel was given together with zymosan A (1 mg/mouse, i.p.) to initiate peritonitis. *For single dosing* of the SPM panel, mice were given vehicle control (0.01% ethanol v/v in 1 mL saline) as control on days 0, 2, 5, 7 and 9. Next, on day 12, the SPM panel was given together with zymosan A (1 mg/mouse, i.p.). Peritoneal exudates were collected at 12, 24 and 48 h, and leukocyte subtypes were assessed using flow cytometry. Aliquots of exudate leukocytes from 12 h time point were used for RNA-seq.

*Resolution indices:* The resolution of acute inflammation was defined in quantitative terms by resolution indices (Ri) as follows. ψ_max_: maximal exudate PMN numbers, T_max_: the time point when PMN numbers reach ψ_max_, R_50_: 50% of ψ_max_, T_50_, the time point when the PMN numbers reached R_50_, R*i* (resolution interval): the time interval from the maximal PMN point ψ_max_ to R_50_, i.e., T_50_ – T_max_, calculated as in Schwab et al. (2007).

### Lipid mediator metabololipidomics

To monitor the production of lipid mediators in mouse exudates, liquid chromatography and electrospray ionization tandem mass spectrometry (LC–ESI–MS/MS) analysis was carried out. Prior to solid-phase sample extraction, cold methanol (Thermo Fisher Scientific, Waltham, MA) held on ice containing deuterated d_4_-LTB_4_, d_8_-5*S*-HETE, d_4_-PGE_2_, d_5_-RvD1, d_5_-RvD2, d_5_-RvD3, and d_5_-LXA_4_ (Cayman Chemical, Ann Arbor, MI) internal standards (500 pg each) were added to each sample. The deuterium-labeled lipid mediators (LM) were initially synthesized to facilitate structural analysis of MS–MS ions, and confirmed the corresponding MS–MS fragmentation mechanisms of these LMs (Hong et al. 2007). These are now commercially available. Here, these deuterated LM were added to each sample to facilitate quantification of recovery from solid-phase extraction. All samples were placed at –80 °C for at least 30 min to allow protein precipitation, followed by solid-phase extraction as detailed earlier in Shay et al. (2021). Next, the extracted samples were subjected to LC–ESI–MS/MS in negative polarity. These experiments were conducted with a QTRAP 6500 + (Sciex, Framingham, MA) equipped with a ExionLC system and a Kinetex® 2.6 μm Polar C18 100 Å, 100 × 3.0 mm column (part no. 00D-4759-Y0, Phenomenex, Torrance, CA) maintained at 50 °C in a column oven. For LC conditions including flow rate, gradient, and mobile phase composition, see Table S1.

Targeted multiple reaction monitoring (MRM) and enhanced product ion (EPI) scan experiments were employed to monitor and quantify lipid mediators of interest. Calibration curves were constructed for each lipid mediator using synthetic and deuterated standard mixtures at, for example, 0.1, 0.5, 1, 5, 10, 25, 50, 125, 250, 500, and 1000 pg ranges, which showed good linearity and gave r^2^ values of 0.98–0.99. Source and gas parameters were set as follows: collision gas (CAD) = 12, curtain gas (CUR) = 30, ion source gas 1 (GS1, psi) = 85, ion source gas 2 (GS2, psi) = 50, ion spray voltage (IS, V) = -4200, and temperature (TEM, ^o^C) = 500. Each mediator was identified by matching its chromatographic retention time (T_R_) and ESI–MS/MS to those of synthetic standards. A custom library containing the individual MS/MS spectra of synthetic and authentic standards was utilized to evaluate spectral matching. Spectral library search parameters were set as follows: precursor mass tolerance ± 0.8 Dalton; collision energy ± 5 eV; use polarity, intensity threshold = 0.05; minimal purity = 5.0%; and intensity factor = 100. Of note, the accuracy for data acquisition of the QTRAP 6500 + is ± 0.1 atomic mass units (a.m.u.). The additional digits presented in MS/MS spectra are due to default manufacturer settings. Data were acquired with Analyst 1.7.1 software (Sciex), and the spectral library was constructed in LibraryView™ software version 1.4.0 (Sciex) in conjunction with Sciex OS-Q v1.7.0.36606 and v3.1.5.3945. Analytes in the exudates that gave a signal-to-noise ratio < 5 or MS/MS library fit score < 70% to the synthetic standard were excluded. Partial least squares-discriminant analysis (PLS-DA) and hierarchical clustering heatmaps were carried out using MetaboAnalyst v6.0 (https://www.metaboanalyst.ca/) (Xia et al. 2009).

### Authentication of RvD1, RvD2, RvD5, RvE2, and MaR1

Synthetic RvD1, RvD5, and RvE2 were purchased from Cayman Chemical (Ann Arbor, MI); RvD2 and MaR1 were from Vinresol (Budapest, Hungary). Prior to use in the present studies, each of these mediators was authenticated using UV spectrophotometry and LC–ESI–MS/MS to assess physical integrity by evaluating chromatographic retention times and MS/MS spectra. These matched the physical properties of the originally identified chemical mediators, were obtained using a Triple Quad 7500 mass spectrometer (Sciex, Framingham, MA) equipped with the ExionLC system (see Table S1) on a Kinetex® 2.6 μm PS C18 100 Å, 100 × 3.0 mm column (part no. 00D-4780-Y0, Phenomenex, Torrance, CA). Source and gas parameters were set as follows: collision gas = 12, curtain gas = 40, ion source gas 1 (psi) = 45, ion source gas 2 (psi) = 70, ion spray voltage (V) = 2000, and source temperature (°C) = 500. Spectral library parameters were set as stated above, and it should be noted that the accuracy for data resolution on the Sciex triple quad 7500 is also ± 0.1 atomic mass units (a.m.u.). Data were acquired and analyzed using Sciex OS 3.1.5.3945 and are presented as screen captures. UV spectra were recorded on a Cary 3500 Compact Peltier UV–visible Spectrophotometer (Agilent Technologies, Santa Clara, CA).

### RNA sequencing and bioinformatics

Mouse exudate leukocytes were collected from peritonitis and submitted for paired-end RNA sequencing using Illumina NovaSeq X (Azenta Life Sciences, Burlington, MA). Sequence reads were trimmed to remove possible adapter sequences and nucleotides with poor quality using Trimmomatic v.0.36. The trimmed reads were mapped to the *Mus musculus* GRCm38 ERCC reference genome available on ENSEMBL using the STAR aligner v.2.5.2b. The STAR aligner is a splice aligner that detects splice junctions and incorporates them to help align the entire read sequences. Unique gene hit counts were calculated by using featureCounts from the Subread package v.1.5.2. Only unique reads that fell within exon regions were counted. The unique gene hit counts were then analyzed using DESeq2, a comparison of gene expression between the groups of samples. The Wald test was used to generate p-values and log_2_ fold changes (Log_2_FC). Genes with a p-value < 0.05 and absolute Log_2_FC > 1 or < -1 were called as differentially expressed genes (DEGs). A gene ontology analysis was performed. The GO list was used to cluster the set of genes based on their biological processes and determine their statistical significance. DEGs were clustered by their gene ontology, and the enrichment of gene ontology terms was tested using Fisher exact test (GeneSCF v1.1-p2). An adjusted P-value less than 0.05 is considered statistically significant. The enrichment effect for each pathway was calculated as follows: Ratio 1 = “Significant_genes_count” in a GO category divided by number of significant gene numbers, which was 82 in our dataset; Ratio 2 = “Total_genes_group_count” in the same GO category divided by the total number of genes in the mouse genome, which was 49,671 in our dataset; Enrichment effect = Ratio 1/Ratio 2. Network analyses were also performed using “Atlas of Inflammation Resolution (AIR)” (https://air.bio.informatik.uni-rostock.de) to better understand the non-linear relationship among immune cell types, signaling and regulatory molecules associated with the onset, transition, resolution of acute inflammation and homeostasis (Serhan et al. 2020; Hoch et al. 2022).

### Human macrophage and receptor expression

Human peripheral blood mononuclear cells were obtained from the Boston Children's Hospital Blood Bank (Mass General Brigham investigational review board protocol #1999-P-001279). All donors are adults. Peripheral blood mononuclear cells were isolated, and MΦ were differentiated by culturing freshly isolated monocytes in RPMI media supplemented with 10% FBS and recombinant human GM-CSF (10 ng/ml; R&D Systems) for 7 d. Macrophages were then incubated with 10 nM of RvD1, RvD2, MaR1, RvE2 or vehicle control for 24 h. Cells were collected and receptor surface expression monitored by flow cytometry using the following specific antibodies and appropriate isotype controls: GPR18 Antibody [Alexa Fluor® 647] (NBP2-24918AF647, Novus Biologicals) and Rabbit IgG Isotype Control [Alexa Fluor® 647] (NBP2-36463AF647, Novus Biologicals), human ChemR23 PE-conjugated Antibody (FAB362P, R&D systems) and mouse IgG3 PE-conjugated Antibody (IC007P, R&D systems), LGR6 Polyclonal antibody (17658-1-AP, Proteintech) and PE-conjugated F(ab')2-Donkey anti-Rabbit IgG (H + L) Secondary Antibody (12-4739-81, eBioscience).

### Statistical analysis

Statistical analyses were performed using 2-tailed Student’s t test for two-group comparisons or one-way ANOVA with multiple group comparisons for three or more independent groups (GraphPad Prism, Version 10.1.0). P values of less than 0.05 were taken as statistically significant.

## Results

### Bacterial challenge during ongoing inflammation shifts resolution indices

**Fig. 1:**
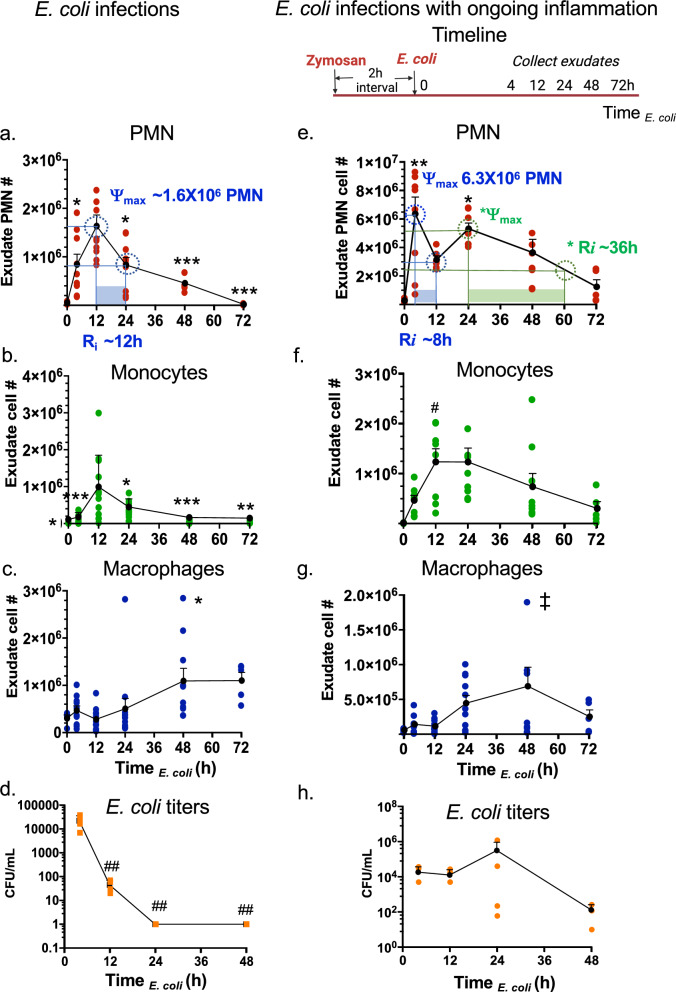
Resolution intervals for *E. coli* infections shift with ongoing zymosan inflammation. (a-d) *E. coli* infection. Mice (C57B6, 6-wk old male) were given saline (1 ml, i.p.) two hours prior to inoculation of *E. coli* (10^5^ CFU, i.p.). Peritoneal exudates were collected by lavaging at indicated time points (Time _*E. coli*_). Total leukocytes were enumerated, and leukocyte composition determined using flow cytometry (see representative dot plots in Supplementary Fig. 1). **a** Exudate PMN time course and resolution indices (ψ_max_ and R*i*, see Methods). **b** exudate monocyte, **c** macrophage and **d** *E. coli* titer time course. **e**–**h** *E. coli* infection with ongoing inflammation. Timeline: Mice were given zymosan (1 mg/ml, i.p.) two hours prior to inoculation of *E. coli* (10^5^ CFU, i.p.). Peritoneal exudates were collected by lavaging at indicated time points (Time _*E. coli*_). **e** Exudate PMN time course and resolution indices (ψ_max_ and R*i*, see Methods). **f** exudate monocyte, **g** macrophage and **h** *E. coli* titer time course. **a**–**c**, **e**–**g** Three independent experiments were carried out and 3-4 mice were used for each time point in each experiment. Results are cell numbers/mouse exudate; mean ± SEM; each dot denotes the value obtained from individual mice. (d,h) One dataset from two independent experiments are shown, and in each experiment, *E. coli* titers of 3-4 mice for each time point were determined. Results are *E. coli* titers/mouse exudate; mean ± SEM; each dot is the value obtained from one mouse. (a-d) *P < 0.05, **P < 0.01, ***P < 0.001 vs 12 h; ^##^P < 0.01 vs 4 h. **e**–**g** *P < 0.05, **P < 0.01 vs. 0 and 72 h; ^#^P < 0.05 vs 0 h; ^‡^P < 0.05 vs 4 h using one-way ANOVA with Tukey's post-test

During self-resolving *E. coli* infectious inflammation, the pro-resolving mediators, i.e., SPMs, are produced and activate endogenous resolution programs (Chiang et al. 2012). Here, we questioned if consecutive challenge with *E. coli* infection would alter the temporal sequence of SPM production and the resolution programs. To address this, we used a well-established murine peritonitis model (Winyard and Willoughby 2003), relevant to human peritonitis (Moore 1959). Inoculation of *E. coli* (10^5^ CFU, *i.p*.) initiated a self-limited innate acute inflammation, characterized by a time-dependent neutrophil infiltration as expected (Fig. 1a and Fig. S1a-b). With these results, we calculated the resolution indices from the present results using equations defined earlier in Schwab et al. (2007). The PMN (CD45 + CD11b + Ly6G + Ly6C-) infiltration into the peritoneum was monitored throughout the time course of the experiments; PMN reached maximum ψ_max_ ~ 1.8 × 10^6^ PMN at 12 h, followed by a rapid decline associated with the resolution response. The resolution interval (R*i*) was 12 h (Fig. 1a). The infiltration of monocytes (CD45 + CD11b + Ly6G-Ly6C +) was initially low in numbers at 4 h (Fig, 1b). These monocytes reached maximum at 12 h, and macrophages (MΦ; CD45 + CD11b + F4/80 +) gradually increased between 12 to 48 h (Fig. 1c). In these experiments, exudate *E. coli* titers were determined and expressed as colony forming units (CFUs). *E. coli* titers (CFUs) were the highest at 4 h, demonstrating robust infection that was cleared by 12 h (Fig. 1d). These results demonstrated a self-resolving *E. coli* infection (Fig. 1a–d). These present results are consistent with our earlier findings (Chiang et al. 2012) that enable us to investigate the impact of sequential challenge.

To gain insight into which of the SPM pathways, i.e., resolvins, protectins or maresins, are most critical to activation of the resolution response, we devised a protocol of ongoing zymosan-induced inflammation followed by *E. coli* infection (Fig. 1e-h). Next, we challenged mice using self-resolving *E. coli* peritonitis with ongoing sterile yeast-derived zymosan (Fig. 1e), a classic inflammatory stimulus (Winyard and Willoughby 2003). With zymosan challenge prior to *E. coli*, PMN infiltration was accelerated with a higher magnitude, reaching maximum ψ_max_ 6.3 × 10^6^ PMN (Fig. 1e and Fig. S1 c-d) following *E. coli* inoculation, compared to ψ_max_ 1.8 × 10^6^ PMN on *E. coli* challenge alone (Fig. 1a). PMN numbers then declined to half ψ_max_ at 12 h, giving a resolution interval (R*i*) of 8 h (Fig. 1e). In these studies, there was a second increase of PMN numbers with *ψ_max_ 5.3 × 10^6^ PMN at 24 h that gradually declined to half *ψ_max_ at ~ 60 h, thus giving a biphasic response with two resolution intervals R*i* = 8 h and *R*i* = 36 h. In these exudates, monocytes reached maximum at 12 h, macrophages continued to increase till 48 h then declined (Fig. 1f and g). Exudate *E. coli* titers reached maximum at 24 h then gradually cleared at 48 h (Fig. 1h). These results demonstrated that a self-resolving *E. coli* peritonitis initiated during ongoing inflammation was reset temporally, thus shifting the exudate leukocyte numbers, *E. coli* clearance, and resolution intervals (Fig. S2).

### Temporal exudate LM-SPM signature profiles

**Fig. 2:**
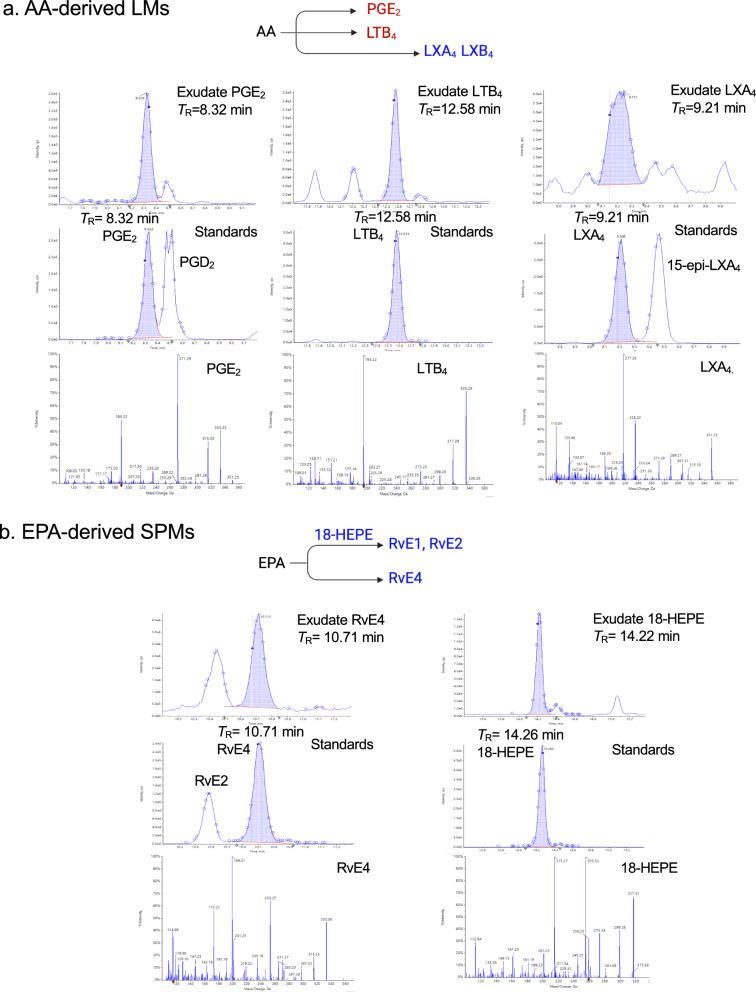

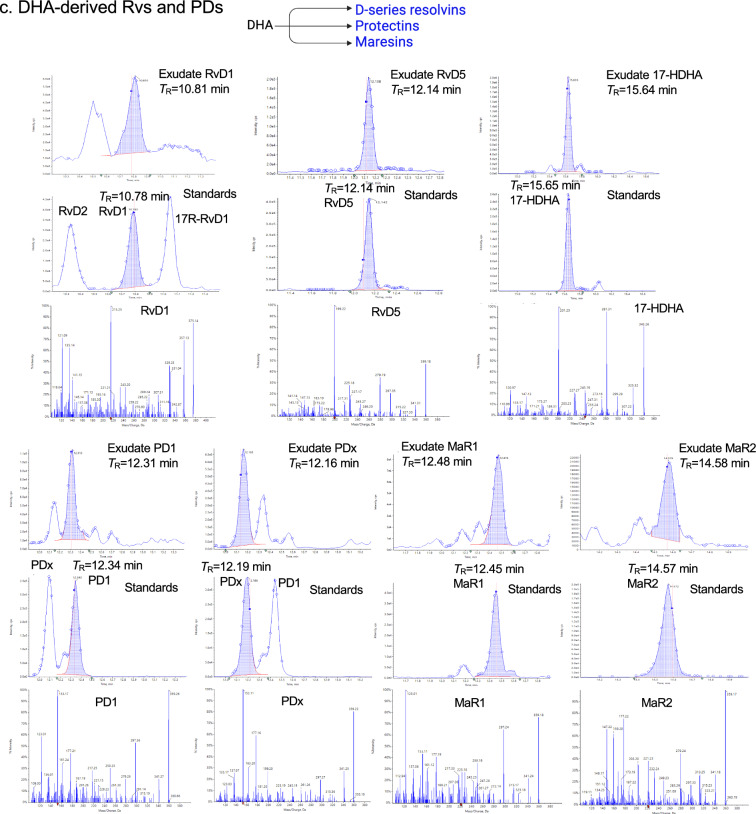
Identification of exudate lipid mediators (LM): SPMs and eicosanoids. Exudate SPMs and eicosanoids were identified using LC–MS-MS-based metabololipidomics (see Methods). Screen captures of MRM chromatograms and MS–MS of identified LMs. For each LM, MRM of exudate LM (top panel) and synthetic standards (middle panel) as well as MS–MS of synthetic standard (bottom panel) are shown. See Fig. S3 for the enlarged MS–MS. The retention time (*T*_R_) of individual LM matched that obtained with synthetic standard. In MRM chromatograms, shaded blue areas denote the area under the curve used for quantitation. Dark blue data points indicate where the spectra were collected. **a** AA-derived LMs. PGE_2_: *T*_R_ 8.32 min, MRM of *m/z* 351 > 189, S/N ratio 643; LTB_4_: *T*_R_ 12.58 min, MRM of *m/z* 335 > 195, S/N ratio 402; LXA_4_: *T*_R_ 9.21 min; MRM of *m/z* 351 > 115; S/N ratio 69. **b** EPA-derived SPMs. RvE4: *T*_R_ 10.71 min, MRM of *m/z* 333 > 115, S/N ratio 159; 18-HEPE: *T*_R_ 14.22 min, MRM of *m/z* 317 > 259, S/N ratio 730. **c** DHA-derived Rvs, PDs, and MaRs. RvD1: *T*_R_ 10.81 min, MRM of *m/z* 375 > 215, S/N ratio 39; RvD5: *T*_R_ 12.14 min, MRM of *m/z* 359 > 199, S/N ratio 332; 17-HDHA: *T*_R_ 15.64 min, MRM of *m/z* 343 > 245, S/N ratio 1,610; PD1: *T*_R_ 12.31 min, MRM of *m/z* 359 > 153, S/N ratio 127; PDx: *T*_R_ 12.16 min, MRM of *m/z* 359 > 153, S/N ratio 409; MaR1: *T*_R_ 12.48 min, MRM of *m/z* 359 > 221, S/N ratio 148; MaR2: *T*_R_ 14.58 min, MRM of *m/z* 359 > 221, S/N ratio 269

Targeted metabololipidomics profiling was carried out with *E. coli* exudates from Fig. 1, using mass spectrometry-based lipid mediator metabololipidomics (see Methods), focusing on local-acting lipid mediators including SPMs (Fig. 2 and S3). For identification of exudate lipid mediators, retention times (*T*_R_) and prominent MS–MS fragmentation ions present on MS–MS for each eicosanoid and SPM were matched to those obtained with both synthetic and authentic standards, which were in accordance with original published criteria for each molecule (Hong et al. 2007; Serhan and Petasis 2011; Serhan and Chiang 2023; Serhan 2014). Screen captures of the MRM chromatograms for each identified mediator in exudates together with those of validated synthetic standards are shown in Fig. 2. In these experiments, lipid mediators identified in exudates included the arachidonic acid-derived PGE_2_, LTB_4_ (both inflammatory (Majno and Joris 2004; Lammermann et al. 2013)) and the resolving mediator LXA_4_ (Serhan 2007), the eicosapentaenoic acid-derived RvE4 and 18-HEPE, as well as the docosahexaenoic acid-derived RvD1, RvD5, PD1, PDx, MaR1 and MaR2. Each of these pro-resolving mediators is a potent agonist of the resolution of inflammation (Serhan 2007; Fredman and Serhan 2024; Schwab et al. 2007).

The MS–MS prominent ions used for identification of eicosanoids and SPMs were as follows and shown in Fig. 2 and S3. After extraction of the peritoneal exudates, targeted LC–MS-MS demonstrated the presence of the arachidonic acid inflammation-resolution metabolome. The MS–MS spectra of exudate LXA_4_ matched those of both synthetic and authentic LXA_4_ (Fig. 2a) and consisted of a parent ion at *m/z* 351 = M-H and ions at *m/z* 315 = M-H-2H_2_O, 307 = M-H-CO_2_, 289 = M-H-H_2_O-CO_2_, 271 = M-H-2H_2_O-CO_2_, 251 = M-H-CHOH-(CH_2_)_4_-CH_3_ + H, 235 = M-H-CHOH-(CH_2_)_3_-CO_2_, 233 = 251-H_2_O, 217 = 235-H_2_O, 199 = 235-2H_2_O, 189 = 251-H_2_O-CO_2_, 135 = M-H-115-CHOH-(CH_2_)_4_-CH_3_, 115 = M-H-CHOH-(CH)_8_-CHOH-(CH_2_)_4_-CH_3_-H. These ions matched those of LXA_4_ documented in Clish et al. (2000). In the eicosapentaenoic acid metabolome: 18-HEPE is a SPM and precursor to E-series resolvins (RvE), uncovered in resolving inflammatory exudates (reviewed in Serhan and Petasis (2011); Serhan (2014)). Also, 18-HEPE is a potent bioactive SPM that, for example, prevents maladaptive cardiac remodeling (Endo et al. 2014). The MS–MS spectra of exudate 18-HEPE matched those of authentic 18-HEPE (Fig. 2b) that gave a parent ion at *m/z* 317 = M-H and daughter ions at *m/z* 299 = M-H-H_2_O, 273 = M-H-CO_2_, 259 = M-H-CHOH-CH_2_-CH_3_ + H, 255 = M-H-H_2_O-CO_2_, 215 = 259-CO_2_. The MS–MS spectra of exudate RvE4 matched those of authentic RvE4 (Fig. 2b) that gave a parent ion at *m/z* 333 = M-H and daughter ions at *m/z* 315 = M-H-H_2_O, 271 = M-H-H_2_O-CO_2_, 253 = M-H-2H_2_O-CO_2_, 235 = CHOH-CH_2_-(CH)_2_-CH_2_-CH_3_ + H, 217 = M-H-CHOH-(CH_2_)_3_-CO_2_, 199 = 217-H_2_O, 173 = 235-H_2_O-CO_2_, 115 = M-H-(CH)_4_-CH_2_-(CH)_4_-CHOH-CH_2_-(CH)_2_-CH_2_-CH_3_-H, and matched those of synthetic validated RvE4 (cf. (Serhan et al. 2022) and references within).

In the inflammatory exudate docosahexaenoic acid metabolome, the MS–MS spectra of exudate RvD1 matched those of authentic synthetic RvD1 (Fig. 2c), which MS–MS fragmentation consisted of a parent ion at *m/z* 375 = M-H and daughter ions at *m/z* 357 = M-H-H_2_O, 305 = M-H-CH_2_-(CH)_2_-CH_2_-CH_3_, 277 = M-CHOH-CH_2_-(CH)_2_-CH_2_-CH_3_ + H, 259 = 277-H_2_O, 243 = 305-H_2_O-CO_2_, 233 = M-H-CHOH-CH_2_-(CH)_2_-(CH_2_)_2_-CO_2_, 215 = 233-H_2_O, 171 = M-H-(CH)_8_-CHOH-CH_2_-(CH)_2_-CH_2_-CH_3_-H, 141 = M-H-CHOH-(CH)_8_-CHOH-CH_2_-(CH)_2_-CH_2_-CH_3_-H, 135 = 171-2H_2_O. These ions matched those of synthetic validated RvD1 documented in Hong et al. (2007). The MS–MS spectra of exudate RvD5 also matched those of synthetic RvD5 (Fig. 2c), consisting of a parent ion at *m/z* 359 = M-H with the daughter ions at *m/z* 341 = M-H-H_2_O, 315 = M-H-CO_2_, 297 = M-H-H_2_O-CO_2_, 289 = M-H-CH_2_-(CH)_2_-CH_2_-CH_3_-H, 279 = M-H-2H_2_O-CO_2_, 246 = M-H-CH_2_-(CH)_2_-(CH_2_)_2_-CO_2_, 227 = 289-H_2_O-CO_2_, 217 = M-H-CHOH-CH_2_-(CH)_2_-(CH_2_)_2_-CO_2_, 199 = 217-H_2_O, 141 = M-H-(CH)_4_-CH_2_-(CH)_4_-CHOH-CH_2_-(CH)_2_-CH_2_-CH_3_-H. These ions matched those of authentic RvD5 documented in ((Hong et al. 2007; Serhan and Petasis 2011; Serhan and Chiang 2023) and original references within) and Chiang et al., 2012 (Chiang et al. 2012). 17-HDHA is a SPM and pathway marker of both D-series resolvins and protectins (Hong et al. 2007). The MS–MS spectra of exudate 17-HDHA matched those of synthetic 17-HDHA (Fig. 2c) that gave a parent ion at *m/z* 343 = M-H with daughter ions at *m/z* 325 = M-H-H_2_O, 299 = M-H-CO_2_, 281 = M-H-H_2_O-CO_2_, 273 = M-H-CH_2_-(CH)_2_-CH_2_-CH_3_-H, 255 = 273-H_2_O, 245 = M-CHOH-CH_2_-(CH)_2_-CH_2_-CH_3_ + H, 229 = 273-CO_2_, 227 = 245-H_2_O, 201 = 245-CO_2_ (Fig. 2c). These fragmentation ions (Fig. 2c) matched those of authentic 17-HDHA reported earlier in Hong et al. (2007).

In the inflammatory exudates, we also identified protectins (PD) and maresins (MaR). The MS–MS spectra of exudate PD1 matched those of authentic and synthetic PD1 (Fig. 2c), which gave a MS–MS parent ion at *m/z* 359 = M-H and daughter ions at *m/z* 341 = M-H-H_2_O, 315 = M-H-CO_2_, 297 = M-H-H_2_O-CO_2_, 279 = M-H-2H_2_O-CO_2_, 261 = M-H-CHOH-CH_2_-(CH)_2_-CH_2_-CH_3_ + H, 217 = 261-CO_2_, 177 = M-H-CHOH-CH_2_-(CH)_2_-CH_2_-(CH)_2_-(CH_2_)_2_-CO_2_, 153 = M-H-CHOH-(CH)_6_-CHOH-CH_2_-(CH)_2_-CH_2_-CH_3_ + H (Fig. 2c). These ions matched those of validated synthetic PD1 as documented in Hong et al. (2007); Serhan and Petasis 2011). PDx is a stereoisomer of PD1 and a double dioxygenation product (Hong et al. 2007; Serhan and Petasis 2011). The MS–MS spectra of exudate PDx matched those of synthetic PDx (Fig. 2c), consisting of a parent ion at *m/z* 359 = M-H and daughter ions at *m/z* 341 = M-H-H_2_O, 315 = M-H-CO_2_, 297 = M-H-H_2_O-CO_2_, 290 = M-H-CH_2_-(CH)_2_-CH_2_-CH_3_, 261 = M-H-CHOH-CH_2_-(CH)_2_-CH_2_-CH_3_ + H, 181 = M-H-(CH)_6_-CHOH-CH_2_-(CH)_2_-CH_2_-CH_3_-H, 177 = M-H-CHOH-CH_2_-(CH)_2_-CH_2_-(CH)_2_-(CH_2_)_2_-CO_2_, 153 = M-H-CHOH-(CH)_6_-CHOH-CH_2_-(CH)_2_-CH_2_-CH_3_ + H, 137 = 181-CO_2_. In the maresin pathway, the MS–MS spectra of MaR1 obtained from exudates matched those of synthetic authentic MaR1 (Fig. 2c). These consisted of the parent ion at *m/z* 359 = M-H and daughter ions at *m/z* 341 = M-H-H_2_O, 315 = M-H-CO_2_, 297 = M-H-H_2_O-CO_2_, 250 = M-H-CH_2_-(CH)_2_-CH_2_-(CH)_2_-CH_2_-CH_3_, 246 = M-H-CH_2_-(CH)_2_-(CH_2_)_2_-CO_2_, 228 = 246-H_2_O, 221 = M-H-CHOH-CH_2_-(CH)_2_-CH_2_-(CH)_2_-CH_2_-CH_3_ + H, 177 = 221-CO_2_, 201 = 221-H_2_O-2H, 123 = 141-H_2_O. These fragmentation ions matched those of validated synthetic MaR1 as documented in (Lau et al. 2023 and original references within). The MS–MS spectra of MaR2 identified in the inflammatory exudates matched those obtained from synthetic MaR2 (Fig. 2c), which consisted of a parent ion at *m/z* 359 = M-H and daughter ions at *m/z* 341 = M-H-H_2_O, 323 = M-H-2H_2_O, 313 = M-H-CO_2_-2H, 297 = M-H-CO_2_-H_2_O, 279 = M-H-2H_2_O-CO_2_, 249 = M-H-CH_2_-(CH)_2_-CH_2_-(CH)_2_-CH_2_-CH_3_-H, 221 = M-H-CHOH-CH_2_-(CH)_2_-CH_2_-(CH)_2_-CH_2_-CH_3_ + H, 203 = 221-H_2_O, 191 = M-H-(CHOH)_2_-CH_2_-(CH)_2_-CH_2_-(CH)_2_-CH_2_-CH_3_ + H, 177 = 221-CO_2_, 167 = M-H-(CH)_6_-CH_2_-(CH)_2_-(CH_2_)_2_-CO_2_-2H, 159 = 221-H_2_O-CO_2_, 147 = 191-CO_2_. These fragmentation ions matched those of MaR2 ((Serhan and Chiang 2023) and original reports within).

**Fig. 3:**
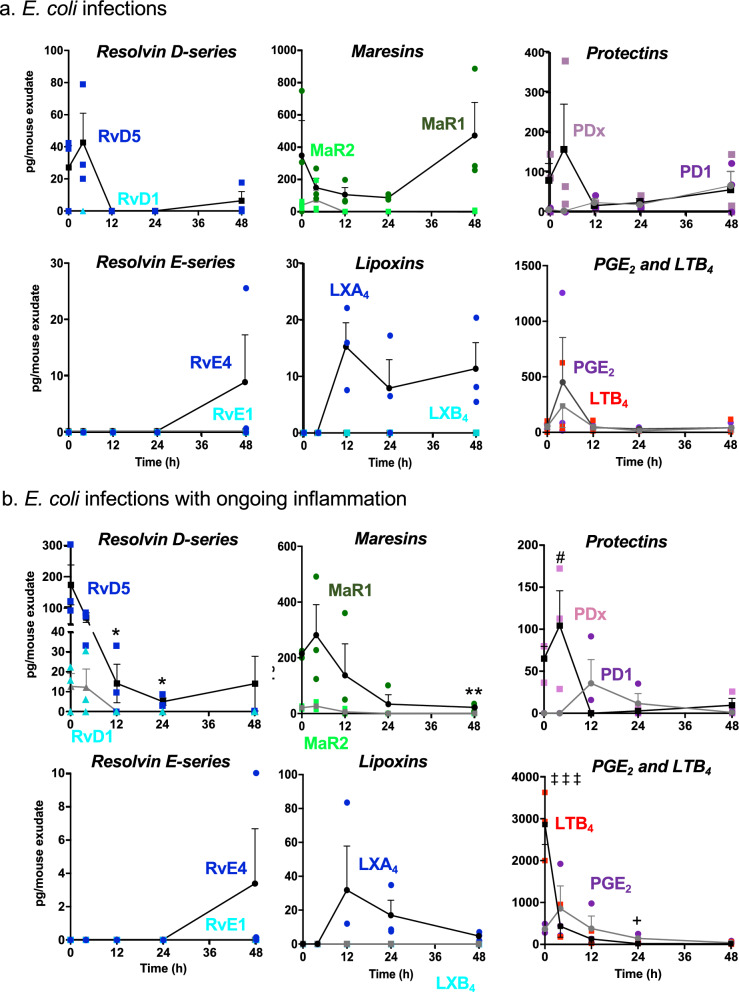
Time course of exudate SPMs and eicosanoids. Exudate SPMs and eicosanoids obtained from mice with **a** *E. coli* infections and **b** *E. coli* infections with ongoing inflammation were each quantified using LC–MS-MS-based metabololipidomics. See Fig. 2 for screen captures of MRM chromatograms of identified SPMs and eicosanoids. Three independent experiments were carried out. In each experiment, 3–4 mouse exudates collected from the same time point were pooled for lipid mediator extraction and LC–MS-MS. Results are expressed as pg/mouse exudate (2 mL); mean ± SEM, each dot denotes the value obtained from one experiment. *P < 0.05 (RvD5), **P < 0.01 (MaR1) vs. 0 h; ^#^P < 0.05 (PDx) vs. 12, 24 and 48 h; ^+^P < 0.05 (PGE_2_) vs. 0 h, ^‡‡‡^P < 0.001 (LTB_4_) vs. 4, 12, 24 and 48 h using one-way ANOVA with Tukey's post-test

During self-resolving *E. coli* infection in the DHA metabolome, RvD5 was present at time 0, increased at 4 h followed by a sharp decline (Fig. 3a and Table S2). By comparison, PD1 gradually increased and reached maximum at 48 h, while levels of PDx, the double dioxygenation product (Serhan 2007) were highest at 4 h. MaR1 was also present at time 0, and gradually reduced between 4-24 h, then reached the highest levels in the resolution phase at 48 h, giving a time course akin to that of macrophages (*cf.* Figure 1c). EPA-derived RvE4 appeared in resolution phase at 48 h, while AA-derived LXA_4_ remained from 12-48 h. Prostaglandin and leukotriene levels were also determined; both PGE_2_ and LTB_4_ reached the highest levels at 4 h (Fig. 3a), preceding maximal PMN infiltration at 12 h (Fig. 1a). Together, these results established the exudate LM temporal profiles in self-resolving *E. coli* peritonitis. These present results are consistent with the lipid mediator class switching (Serhan 2007; Liu et al. 2019). The lipid mediator class switch defines the temporal appearance and relation of pro-inflammatory lipid mediators such as PGE_2_ and LTB_4_ and their time-dependent switch in LM classes to the pro-resolving LMs, e.g. LXA_4_, resolvins, protectins and maresins (Serhan 2007).

We next questioned whether a sterile stimulus zymosan altered these SPM and eicosanoid profiles during *E. coli* infection. Upon zymosan challenge, select SPMs are rapidly generated, including RvD1, RvD5, MaR1 and PDx (Fig. 3b); RvD5 and MaR1 reached the highest levels at early inflammation phase, i.e. 0-4 h after *E. coli* inoculation (T_*E. coli*_; see Timeline in Fig. 1), and remained in the exudates in the late resolution phase at 48 h (Fig. 3b and Table S3). In comparison, PD1 and LXA_4_ reached the maximum at T_*E. coli*_ 12 h then gradually declined. LTB_4_ levels quickly reached maximum upon zymosan challenge T_*E. coli*_ 0 h, while PGE_2_ levels peaked at T_*E. coli*_ 4 h (Fig. 3b), coinciding with maximal exudate PMNs (Fig. 1e).

**Fig. 4:**
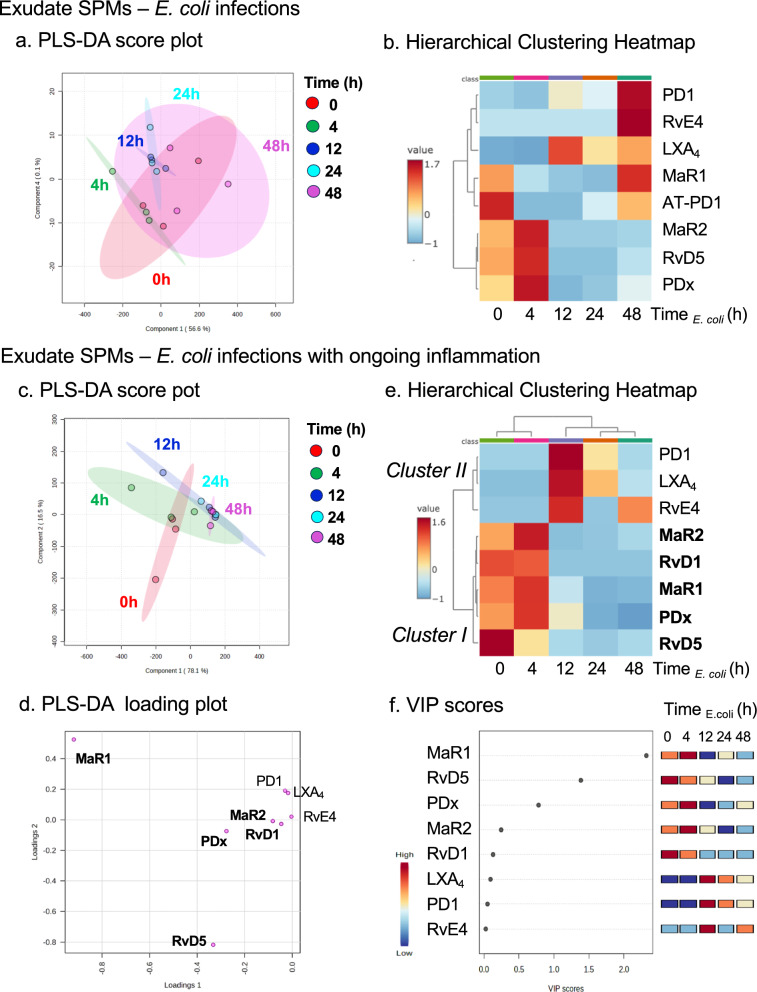
Exudate SPM profiles and clusters on sequential activation. PLS-DA and heatmaps of **a**, **b** *E. coli* infections and (c-f) *E. coli* infections with ongoing inflammation. **a**, **c**, **d** Partial Least Squares Discriminant Analysis (PLS-DA) of identified SPMs. **a**, **c** The score plot (each dot represents profiles from each time point in each experiment) shows clustering among groups (i.e., time points 0, 2, 12, 24 and 48 h), where closer clusters present higher similarity in the data matrix; **d** the loading plot demonstrates correlations in which the measured SPMs contribute to the cluster separation in the score plot. **b**, **e** The hierarchical clustering heatmaps were generated using normalized data with autoscale features. Euclidean distance was used for distance measure and Ward's method was applied in hierarchical cluster analysis (see Methods). Averages of n = 3 for each SPM in each time point are shown. **f** Variable Importance in Projection (VIP) score plot of exudate SPMs, depicting the relative levels of each mediator across the 5 experimental groups (0, 4, 12, 24 and 48 h). RvD5 and MaR1 give highest VIP scores > 1. The colored boxes on the right indicate the relative concentrations (red: high, blue: low) of the corresponding SPMs in each group

PLS-DA was carried out (Fig. 4 and S4) with the identified SPMs in exudates. In *E. coli* infections with ongoing inflammation, the score plot (Fig. 4c) showed distinct separation of different time points from T_*E. coli*_ 0-48 h. The loading plot (Fig. 4d) showed correlations where the measured SPMs contributed to the cluster separation in the score plot. For example, in the early time points following sequential challenge, T_*E. coli*_ 0 h (red) and 4 h (green) clusters were correlated with select SPMs including RvD5 and MaR1 (Fig. 4c, d). The hierarchical clustering Heatmap (Fig. 4e) further indicated clear separation of two SPM clusters: RvD1, RvD5, MaR1, MaR2 and PDx were associated with early time points 0 and 4 h, denoted *Cluster I*; PD1, LXA_4_ and RvE4 are associated with later time points 12, 24 and 48 h, denoted *Cluster II* (Fig. 4e). The Variable Importance in Projection (VIP) scores obtained by PLS-DA showed highest scores for MaR1 and RvD5 in the SPM cluster I (Fig. 4f).

We also carried out PLS-DA with identified exudate SPMs and eicosanoids including LTB_4_ and PGE_2_, which also gave distinct separation of different time points from 0-48 h, as shown in the score plot (Fig. S4). With ANOVA, MaR1, MaR2, RvD5, PDx, 17-HDHA, 18-HEPE, and LTB_4_ were regulated in a temporal manner from 0-48 h that was statistically significant (P < 0.05; Fig. S4a). Hierarchical Clustering Heatmaps (Fig. S4b) also showed two distinct clusters: (Cluster I) PGE_2_, LTB_4_, 18-HEPE, 17-HDHA, RvD1, RvD5, MaR1, MaR2 and PDx associated with early time points 0 and 4 h, and (Cluster II) PD1, LXA_4_ and RvE4 associated with later time points 12, 24 and 48 h. Together, the metabololipidomics results identified select SPM clusters temporally activated when the host was challenged with *E. coli* infection during ongoing zymosan-induced inflammation.

### Very low dose SPMs program the resolution response in vivo

Since zymosan-induced inflammation with subsequent *E. coli* infections initiated production of a SPM cluster in vivo within the infectious exudates (Fig. 4), we set out to investigate whether repetitive dosing of these SPMs would impact the resolution response. We selected a SPM panel that included RvD1, RvD5 and MaR1 because they were identified in the SPM Cluster I. We also included RvD2 and RvE2 in this panel because RvD2 exhibits potent actions increasing survival as we found earlier in sepsis models (Spite et al. 2009), and RvE2 limits PMN infiltration in sterile inflammation (Serhan and Chiang 2023). SPMs in this panel accelerate resolution of acute inflammation, shortening resolution intervals. For example, in self-resolving *E. coli* infection reported in Chiang et al. (2012), RvD1 at 50 ng/mouse shortens R*i* by ~ 40%. With zymosan peritonitis, earlier results (Arnardottir et al. 2016) showed that RvD2 and MaR1 at 50 ng/mouse each shortened R*i* by ~ 75%. In the present experiments, we first carried out zymosan-initiated murine peritonitis where mice were given an SPM panel consisting of RvD1, RvD2, RvD5, MaR1 and RvE2, i.p. at 0.1, 1 or 10 ng each, compared to vehicle control, to emulate the consequence of daily SPM dosing in humans as in Möller et al. (2023); Ramirez et al. 2019). These SPMs were authenticated prior to experiments (Fig. S5); physical properties of each synthetic SPM were examined and compared to published criteria for authentication (Chiang et al. 2012; Serhan and Petasis 2011; Serhan and Chiang 2023; Spite et al. 2009). This SPM panel at dose of 0.1 ng each reduced exudate PMN ~ 25% at 12 h, albeit did not reach statistical significance (Fig. S6). At 1 or 10 ng each, this SPM panel reduced ~ 45 and ~ 65% exudate PMN, respectively (P < 0.05; Fig. S6).

**Fig. 5:**
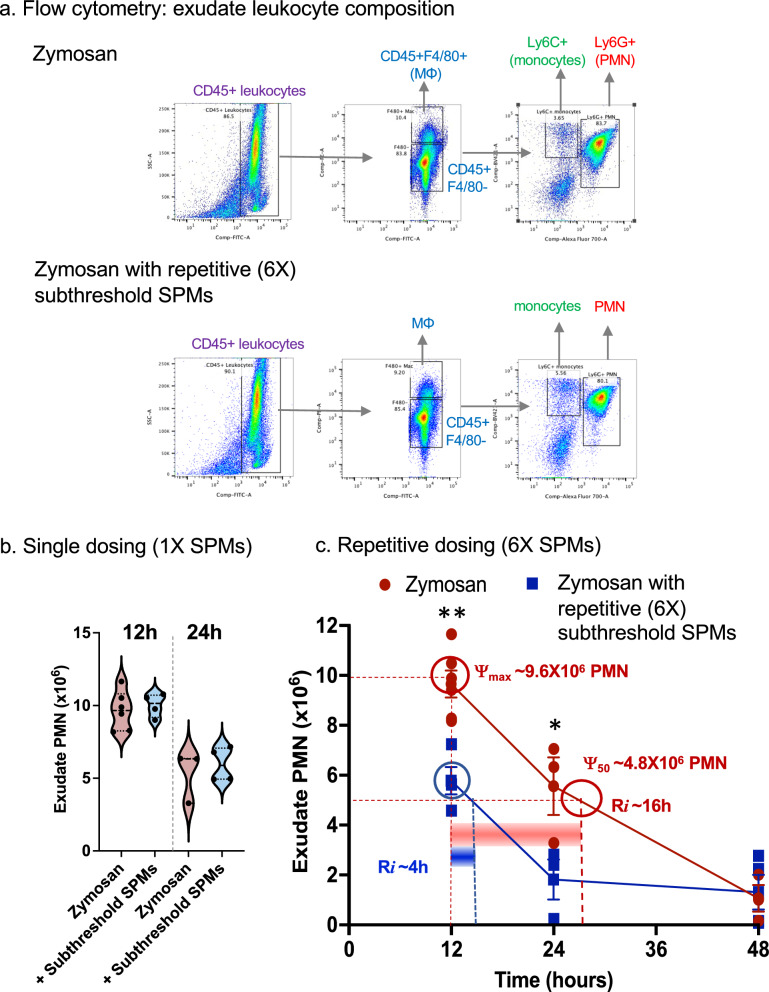
SPM programming in vivo accelerates resolution of inflammation, shortening resolution intervals. Mice were administered with a panel of SPMs (RvD1, RvD2, RvD5, MaR1 and RvE2, 0.1 ng of each SPM in 1 mL saline for each mouse, i.p.) or vehicle control (0.01% ethanol in 1 mL saline) 5 times on Day 0, 2, 5, 7, and 9. On day 12, the panel of SPMs or vehicle was given together with zymosan (1 mg/mouse, i.p.), and exudates collected at indicated time points (see Timelines in Fig. S7a). Total leukocytes were enumerated, and leukocyte composition determined by flow cytometry. **a** Exudate leukocyte composition identified using flow cytometry. Representative dot plots of exudate samples collected at 12 h with gating strategy to identify PMN, monocytes and macrophages. (Top) Zymosan (Bottom) Zymosan with repetitive subthreshold SPMs. **b** Zymosan vs zymosan plus one-time (1X) subthreshold SPMs at 12 and 24 h. **c** Zymosan (red curve) vs. repetitive (6X) subthreshold SPMs (blue curve); time course 12-48 h and resolution indices (ψ_max,_ ψ_50_ and R*i*, see Methods); mean ± SEM, n = 4–6 (12 h) or 3 (24 or 48 h), *P < 0.05, **P < 0.01 using two-tailed Student’s t-test. Each dot denotes cell number obtained from one mouse

Next, we carried out consecutive treatments with this SPM panel at subthreshold doses (RvD1, RvD2, RvD5, MaR1 and RvE2, 0.1 ng each) for 5 times from days 0–12. On day 12, the subthreshold SPM panel or vehicle control was given together with zymosan to initiate peritonitis. See the timelines of single (1X) and repetitive (6X) SPM dosings in Fig. S7a. At 12–48 h, peritoneal exudates were collected, and cellular composition determined by flow cytometry (see gating strategy and leukocyte compositions in Fig. 5a and Table S4). At 12 and 24 h, a single dose of subthreshold SPMs did not reduce exudate PMN in a statistically significant manner (Fig. 5b). Repetitive SPMs reduced exudate PMN by ~ 45% (P < 0.01) at 12 h, and ~ 60% (P < 0.05) at 24 h (Fig. 5c). Resolution indices were calculated. At 12 h, ψ_max_ was ~ 9.6 × 10^6^ PMN, that reduced to 50% at ~ 28 h, giving a resolution interval R*i* ~ 16 h. With repetitive subthreshold SPMs, R*i* markedly reduced 75% to only ~ 4 h. The repetitive SPMs did not significantly alter percentages of exudate macrophages (Table S4). These results indicated that low-dose SPM repetitive dosing regimen accelerated resolution of acute inflammation, as evidenced by shortening the resolution interval (Fig. 5c).

RNA-sequencing was carried out with inflammatory exudates collected at 12 h to determine whether select genes and pathways are regulated by the SPM dosing regimen prior to zymosan exposure in vivo. A total of 14,440 transcripts were obtained from inflammatory exudates. There were 57 known transcripts up-regulated (*P* < 0.05) by repetitive SPM dosing with a fold change (FC) > 2 (log_2_FC > 1), listed in Fig. S8a. Among them, *il-11* (230% increase) is known to have anti-inflammatory properties (Yu et al. 2016); metalloproteinase (*mmp)-2* (130% increase) and *mmp-3* (180% increase), known to have protective roles in chemokine cleavage (Westermann et al. 2011). The transcripts for SPM receptors (reviewed in Serhan and Chiang (2023)) were present including *Fpr2* (a LXA_4_ and RvD1 receptor), *gpr18* (a RvD2 receptor) and *Cmklr1* (a RvE1 and RvE2 receptor) (see Table S5). The *gpr18* transcript was increased by SPM repetitive dosing by ~ 28% (Log_2_FC 0.35), albeit they did not reach statistical significance (Table S5). The transcripts for SPM biosynthesis enzymes are also present but did not appear to be regulated in these exudates (Table S5); these include *Alox15* (12/15-lipoxygenase), *Alox5* (5-lipoxygenase), *Ptgs2* (cyclooxygenase) and *Lta4h* (LTA_4_ hydrolase) (Haeggström and Newcomer 2023). SPM dosing down-regulated select transcripts; 46 known transcripts were down-regulated (*P* < 0.05) with log_2_FC < -0.6 (Fig. S8b).

**Fig. 6:**
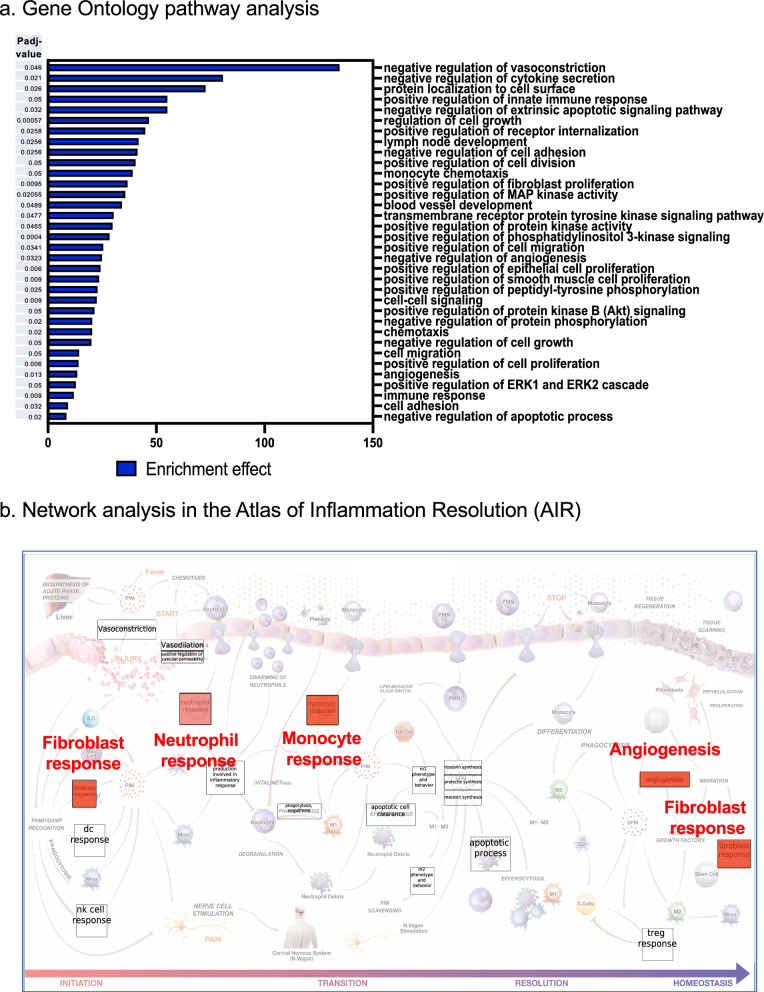
SPMs regulate transcriptome and immune pathways towards resolution and homeostasis. **a** RNA-seq Gene ontology (GO) enrichment analysis of DEGs that are significantly regulated by SPMs, and related to immune functions. X-axis represents enrichment effect (see Methods for the equation) and Y-axis represents different GO terms. The adjusted p-value (Padj-value) are shown on the left for the corresponding GO term when comparing zymosan plus subthreshold SPMs vs. zymosan alone (n = 4), Padj- < 0.05. Also see Supplementary Table S6 for the genes in each GO term. **b** Inflammation-resolution network analysis using “Atlas of Inflammation Resolution (AIR)” (Serhan et al. 2020; Hoch et al. 2022) (https://air.bio.informatik.uni-rostock.de). Inflammation-resolution processes and phenotypes were grouped into 4 phases (inflammation initiation, transition, resolution, and homeostasis). Repetitive subthreshold SPMs up-regulated select phenotypes in each phase, highlighted in red. See Fig. S9a for a clear view of all processes and phenotypes, genes in each phenotype in “Atlas of Inflammation Resolution”, and Fig. S9b for central regulatory network (CRN) for some of these pathways

Gene ontology analysis demonstrated > 300 pathways that were regulated by SPM repetitive dosing (adjusted P values < 0.05). The top 34 pathways related to immune functions are shown in Fig. 6a; some of them are known to have a critical role in the resolution of inflammation and return to homeostasis. Table S6 lists the genes in each significantly regulated GO terms; these include negative regulation of vasoconstriction, cytokine secretion and cell adhesion; positive regulation of innate immune response, cell division, fibroblast and epithelial cell proliferation, and monocyte chemotaxis (Fig. 6a). We next employed Inflammation-resolution network analysis using “Atlas of Inflammation Resolution (AIR)” (https://air.bio.informatik.uni-rostock.de). Inflammation-resolution processes and phenotypes were grouped into 4 phases (inflammation initiation, transition, resolution, and homeostasis), shown in Fig. 6b. SPMs up-regulated select phenotypes in each phase, highlighted in red. Also see Supplementary Figure S9 for SPM-regulated genes in each “phenotype” in the “Atlas of Inflammation Resolution (AIR)”, and a central regulatory network (CRN) representing the molecular interaction associated with the selected phenotype element (e.g., neutrophil, monocyte and fibroblast response). Together, these results using both GO pathway and AIR network analyses indicated that subthreshold SPM dosing in vivo upregulated specific pathways involved in the resolution of inflammation, tissue repair and homeostasis.

To test this principle with human tissue, human monocyte-derived macrophages were incubated with RvD1, RvD2, MaR1 or RvE1 at 10 nM each separately, and surface expression of SPM receptors examined using flow cytometry. These include RvD2 receptor DRV2, MaR1 receptor LGR6 and RvE1 receptor ERV1 (reviewed in Serhan and Chiang (2023)). As shown in Table 1, RvD2 and MaR1 at 24 h each up-regulated (~ 25%, P < 0.01) surface expression of the RvD2 receptor, i.e., DRV2/GPR18. RvD2 also increased surface expression of the MaR1 receptor LGR6 (P < 0.05; Table 1). Under the same conditions, surface expression of ERV1/ChemR23, the receptor for RvE1, was not altered by these SPMs in a statistically significant manner. These findings suggest that the exposure to pro-resolving mediators selectively up-regulates SPM receptors, as a potential feed forward resolution response to accelerate the return to homeostasis.

**Table 1:** Regulation of SPM receptors with human monocyte-derived macrophages

Upregulation of receptor		RvD1	RvD2	MaR1	RvE2
DRV2/GPR18	RvD2	19.8 ± 7.2% (4)	**26.5 ± 3.4% (6)	**24.8 ± 4.9% (6)	20.5 ± 11.0% (6)
LGR6	MaR1	2.8 ± 3.5% (5)	*11.2 ± 3.8% (5)	11.2 ± 7.5% (5)	2.2 ± 4.0% (5)
ERV1/ChemR23	RvE1 & E2	4.4 ± 1.7% (4)	0.0 ± 1.8% (4)	2.9 ± 4.4% (4)	0.0 ± 3.4% (4)

Human macrophages were incubated with 10 nM of RvD1 RvD2, MaR1, RvE2 or vehicle (0.1% ethanol) for 24 h. Cells were collected, and surface expression of GPR18, LGR6 and ChemR23 was determined using flow cytometry. See Methods for the specific antibodies used for these receptors. Results are percent increase of receptor surface expression above vehicle control; mean ± SEM from 4–6 separate donors, and N numbers are denoted within the brackets. *P < 0.05, **P < 0.01 compared to vehicle control using two-tailed Student’s t-test

## Discussion

In the present manuscript, we report that *E. coli* infections encountered during ongoing inflammation reset the temporal resolution response, and that low-dose SPM repetitive regimen re-programs and up-regulates the endogenous resolution mechanisms. Our challenge protocol (Fig. 1e) enabled us to identify two main SPM clusters that were temporally programmed (Figs. 1, 2, 3 and 4). These proved to be RvD1, RvD5, MaR1, MaR2, PDx in Cluster I, and PD1, LXA_4_, RvE4 in Cluster II, uncovered using LC–MS–MS-based identification (Shay et al. 2021) with Partial Least Squares Discriminant Analysis (PLS-DA) in Fig. 4.

The production of SPMs such as D-series resolvins and protectins is a highly conserved system in evolution (Hong et al. 2007) supported by the identification and biosynthesis of RvD1, RvD5 and neuroprotectin D1 in brain cells (Hong et al. 2005) and head kidney gland of the rainbow trout (*Oncorhynchus mykiss*) (Hong et al. 2007). Recently, the head kidney gland cells of the Atlantic Salmon (*Salmon salar*), which is the hematopoietic organ, were found to biosynthesize nanogram amounts of the D-series resolvins (Araujo et al. 2023). These findings suggest that the conserved SPM molecules are essential to the host defense system, and thus can be important to consider in human resilience (Taylor et al. 2022). Norris et al. (2018) found that supplementation of healthy adults with ω-3 DHA and EPA on LPS challenge gives a plasma SPM cluster that consists of RvE1, RvD1, AT-LXA_4_, LXB_4_, 18-HEPE and 17-HDHA. Coagulation of human blood ex vivo initiates temporal production of a SPM cluster that consists of RvD1, RvD5, MaR1, RvE1 and LXB_4_ (Norris et al. 2017). These pro-resolving lipid mediator clusters from earlier human studies (Norris et al. 2018, 2017) share RvD1, RvE1 and LXB_4_ pro-resolving mediators. Results in Fig. 4 of the present study identified RvD1, RvD5, MaR1, MaR2 and PDx (Cluster I) production in mice with ongoing inflammation challenged with *E. coli*. Repetitive low dose (0.1 ng each per mouse) of RvD1, RvD2, RvD5, MaR1 and RvE2 reset the resolution response in vivo.

In humans, SPMs are present in breast milk (Arnardottir et al. 2016), cerebrospinal fluid (Wang et al. 2015), and peripheral blood (Barden et al. 2024; Lau et al. 2023). SPMs increase with omega-3 supplementation (Mas et al. 2012). SPM-containing supplements are increasingly appearing, e.g. SPM-enriched marine oil capsules are commercially available (Möller et al. 2023; Souza et al. 2020) and in daily use, as is the EPA derivative Icosapent Ethyl (Verma et al. 2021) for specific clinical indications. This raises the possibility that daily SPM might down-regulate the endogenous resolution response. On the contrary, here in the present experiments, we found that low-dose SPM in repetitive administration accelerated resolution in this experimental setting. The low-dose SPMs used herein (0.1 ng each) were within the ranges of their quantities present in resolving exudates (Fig. 3 and Table S3). For example, RvD5 range was 64–173 pg and MaR1 214–281 pg per exudate at 0–4 h following sequential challenges, thus in the range of the 100 pg of each SPM per mouse used in these add-back experiments. These low-dose SPMs regulated genes and pathways associated with inflammation and tissue repair (Fig. 6 and S8 and 9). Select SPMs (RvD2 and MaR1) in this panel also significantly regulated SPM receptors present on human monocyte-derived macrophages (Table 1), demonstrating a potential physiologic feed-forward mechanism in resolution.

Along these lines, treatment with a pro-resolving stable mimetic proved to be both safe and effective in oral inflammation (Hasturk et al. 2021). Topical application of the SPM mimetic increased endogenous SPM production documented in vivo in human peripheral blood (Hasturk et al. 2021). Human macrophages are central to resolution and undergo lipid mediator class switch, namely the temporal change in LM classes from the initiation to resolution phase of the acute inflammatory response defined in Serhan (2007) with appropriate stimuli (Liu et al. 2019). EPA and DHA are converted by M2-like macrophages that are a major tissue resource of SPMs (Recchiuti et al. 2014). These SPMs stimulate resolution of inflammation in vivo in experimental animal disease models ((Leroy et al. 2023), reviewed in Serhan and Chiang (2023)). Resolvins also display anti-depressant actions in the brain that are receptor-mediated (Deyama et al. 2018) and evoke brain neurochemical changes in serotonin, dopamine and glutamate (Klein et al. 2014). The nervous system is wired to regulate inflammation via the inflammatory reflex (Diamond and Tracey 2011) that activates resolution mechanisms by stimulating production of resolvins (Mirakaj et al. 2014).

Our present results demonstrate that the resolution response is preemptively activated by both low-dose and repetitive SPM treatment of the innate immune system. Resolvin analogs have been prepared (Murakami et al. 2020) that are highly potent, showing femtomolar activities, which is very promising for therapeutic development (Serhan and Chiang 2023). The activation of SPM receptors stimulates an entire signaling network of anti-inflammation that leads to complete resolution (Suchitha et al. 2024) and activates mesenchymal stem cells (AlZahrani et al. 2022). Our results from RNA-seq with SPMs confirmed and further identified molecules and mechanisms regulated by SPMs. Results in Fig. S8a demonstrated that *il11*, *mmp2/3* and *cx3cl1*/fractalkine are up-regulated by SPMs (Log_2_FC > 1, P < 0.05, Fig S8a). IL-11 is protective in ischemia–reperfusion injury (Yu et al. 2016), colitis (Nishina et al. 2023), and accelerates wound healing (Singh et al. 2023). MMP-2 reduces inflammation via cleaving chemokine such as MCP-3 (Westermann et al. 2011). The fractalkine and its receptor CX3CR1 are protective in liver fibrosis (Karlmark et al. 2010). Along these lines, RvD2 in murine model of sepsis increases MMP-2 and MMP-3 in infectious exudates ((Serhan and Chiang 2023) and references within). With human macrophages, select cysteinyl-conjugated SPMs (cys-SPMs), i.e., Maresin conjugate in tissue regeneration 3 (MCTR3) and Resolvin conjugate in tissue regeneration-3 (RCTR3), increase IL-11 in ((Serhan and Chiang 2023) and references within). Of note, in the present experiments, the low-dose SPMs up-regulated *Kif21a* (Kinesin Family Member 21A; Log_2_FC 1.17, Fig. S8a) and *Lyst* (Log_2_FC 0.45). These genes are also up-regulated by cys-SPMs, MCTR3 and RCTR3 during planaria regeneration uncovered using RNA-seq (Serhan and Chiang 2023 and references within). Lyst (Lysosomal Trafficking Regulator) is a paralog of WDFY3, which is a regulator of macrophage efferocytosis (Shi et al. 2022). The transcription factor *spic* was down-regulated by SPMs (Fig. S8b); spic (Spi-C Transcription Factor) is up-reregulated in Secondary hemophagocytic lymphohistiocytosis, a hyperinflammatory state in humans (Wang et al. 2019). Thus, these effector functions uncovered in the present experiments (Fig. S8) are in line with the SPM pro-resolving mechanisms (Serhan and Chiang 2023; Serhan 2014). Recently, a resolving neutrophil subset was uncovered that produces RvD1 (Geng et al. 2024), that now joins the cellular and molecular mechanisms in the resolution response (Serhan 2007; Fredman and Serhan 2024; Schwab et al. 2007). Both *E. coli* and zymosan activate toll-like receptors (TLR)-2 and TLR4, and their intracellular signaling is blocked by resolvins (Croasdell et al. 2016). Trained immunity and tolerance are orchestrated in the innate immune system to a large extent by TLRs (Divangahi et al. 2021). TLR agonism in sequential infection challenges yield hyperinflammation state (Wang et al. 2019). Our present results demonstrated that (A) the TLR sequential agonism can reset the resolution programs, and (B) the resolution response is trained by low dose repetitive SPM treatment.

## Conclusions

MaR1 (Jiang et al. 2023), RvD1 (Ferri et al. 2023) and RvD2 (Zhang et al. 2016; Hellmann et al. 2018) have proven to play critical functions controlling inflammation in vivo in animal models since their elucidation originally reported in 2002 and 2009 (reviewed in Serhan and Chiang (2023)) and references within). The potent actions of RvD2 (Spite et al. 2009) are promising in controlling infectious inflammation that occurs in sepsis and tissue injury (Sundarasivarao et al. 2022; Padovani and Yin 2024). These earlier findings and results of the present study provide evidence of positive feed-forward mechanisms in the resolution response evoked by SPM repetitive low-dose therapy that has implications for their long-term use and clinical development. In the present experiments, we used a protocol of infection with underlying inflammation with the goal of identifying which of the superfamily of SPMs are produced and essential to stimulate resolution of infectious inflammation. Importantly, the panel of SPMs that include RvD1, RvD2, RvD5, MaR1 and RvE2 reset the temporal cellular and molecular events in the resolution response. Given in low doses, these SPMs reprogrammed the start sequence of the resolution response code. Together, these findings in mice have direct implications for preventative medicine and resolution pharmacology (Serhan 2014). These may be useful in preparing for the next pandemic (Huang et al. 2024; Morse et al. 2012), particularly in individuals with ongoing inflammation as in arthritis, cardiovascular and neurodegenerative diseases that are co-morbidities in viral pandemics such as COVID-19 by raising resilience, via trained SPM resolution response. These studies warrant further investigation and translation to humans to assess the SPM trained resolution response and nutritional maintenance.

## Supplementary Information

Supplementary Material 1.

## Data Availability

RNA-seq and LC–MS-MS datasets generated and analyzed in the current study will be available on publication in the repositories: Gene Expression Omnibus (https://www.ncbi.nlm.nih.gov/geo/) GEO accession no.GSE270972 and BioStudies (https://www.ebi.ac.uk/biostudies/) accession S-BSST1467, respectively.

## Abbreviations

AA: Arachidonic acid
AIR: Atlas of inflammation resolution
CRN: Central regulatory network
DEG: Differentially expressed genes
DHA: Docosahexaenoic acid
EPA: Eicosapentaenoic acid
LC–MS/MS: Liquid chromatography tandem mass spectrometry
EPI: Enhanced product ion
ESI: Electrospray ionization
LM: Lipid mediators
LT: Leukotriene
LX: Lipoxin
MΦ: Macrophage
MaR: Maresin
MMP: Metalloproteinase
MRM: Multiple reaction monitoring
PD: Protectin
PLS-DA: Partial least squares-discriminant analysis
PG: Prostaglandin
PMN: Polymorphonuclear neutrophil
RI: Resolution indices
ψ_max_: Maximal exudate PMN numbers
T_max_: The time point when PMN numbers reach ψ_max_
R_50_: 50% Of ψ_max_
T_50_: The time point when the PMN numbers reached R_50_
R*i* (resolution interval): The time interval from the maximal PMN point ψ_max_ to R_50_, i.e., T_50_−T_max_
Rv: Resolvins
SPMs: Specialized pro-resolving mediators
*T*_R_: Retention time
