# Supplementary material for: Low-dose pro-resolving mediators temporally reset the resolution response to microbial inflammation
Source: Mol Med. 2024 Sep 18;30:153. doi: 10.1186/s10020-024-00877-w (PMC11411770; doi:10.1186/s10020-024-00877-w)
Supplement: Supplementary file 1 — Supplementary Material 1. [file 10020_2024_877_MOESM1_ESM.pdf]

Supplementary Material

Low-dose Pro-resolving Mediators Temporally Reset the Resolution Response to Microbial Inflammation

Charles N. Serhan, Nan Chiang and Robert Nshimiyimana

Center for Experimental Therapeutics and Reperfusion Injury, Department of Anesthesiology, Perioperative and Pain Medicine, Mass General Brigham and Harvard Medical School, Boston, Massachusetts 02115, USA

Address correspondence and reprint requests to:

Prof. Charles N. Serhan, Director

Center for Experimental Therapeutics and Reperfusion Injury,

60 Fenwood Rd., Hale Building for Transformative Medicine 3-016, Boston, Massachusetts 02115, USA.

Phone: 617-525-5001; Fax: 617-525-5017

E-mail: cserhan@bwh.harvard.edu

ORCID:

Charles N. Serhan: 0000-0003-4627-8545

Nan Chiang: 0000-0003-1963-1585

Robert Nshimiyimana: 0000-0003-0832-9938

a. Timeline – *E. coli* challenge

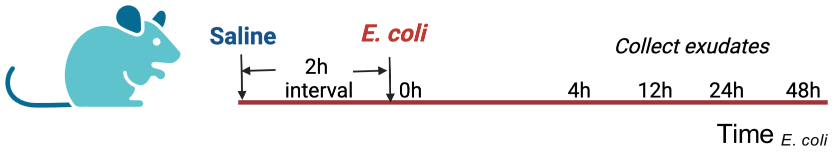

b. *E. coli* challenge –
Exudate leukocyte populations: representative flow cytometry.

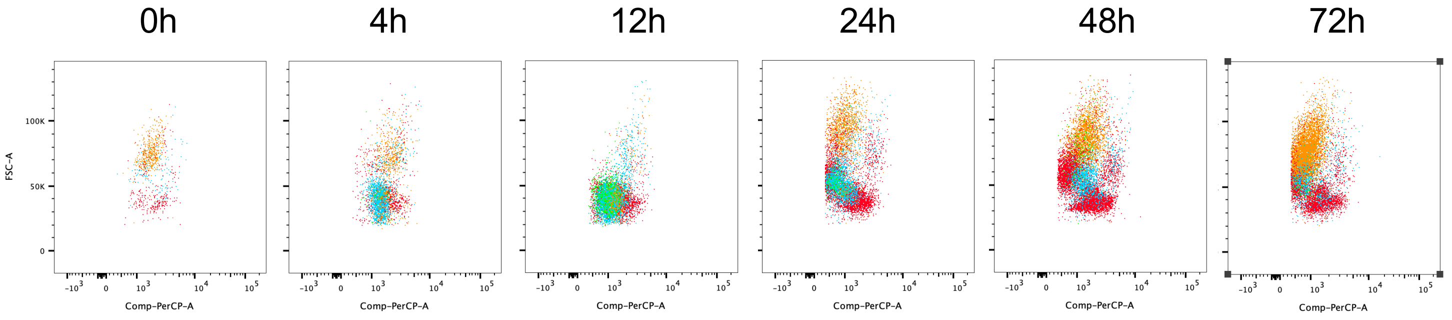

c. Timeline –*E. coli* challenge with ongoing inflammation

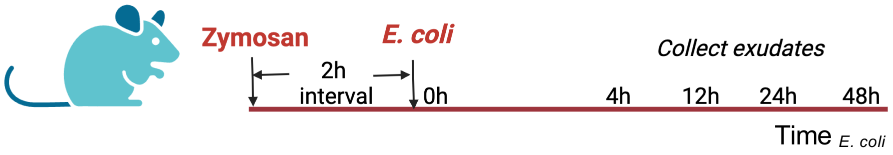

d. *E. coli* challenge with ongoing inflammation -
Exudate leukocyte populations: representative flow cytometry.

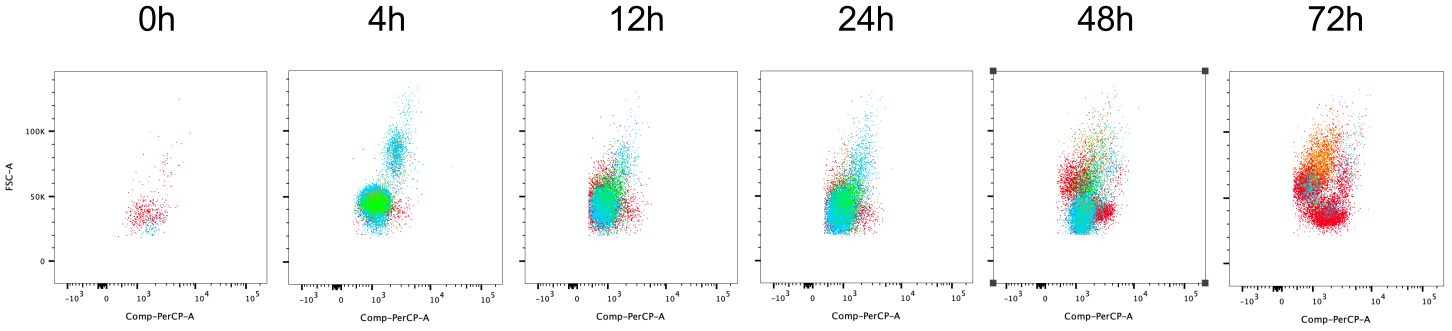

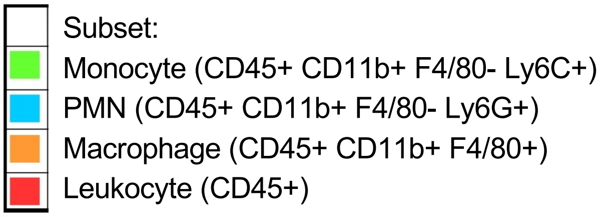

Supplementary Figure S1. Self-resolving *E. coli* infection with or without ongoing inflammation: timelines and flow cytometry

(a) Timeline. Mice (C57B6, 6-wk old male) were given saline (1 ml, i.p.) two hours prior to inoculation of *E. coli* (10^5 CFU, i.p.). Peritoneal exudates were collected by lavaging at indicated time points. Total leukocytes were enumerated and leukocyte composition determined using flow cytometry.

(b) Representative flow cytometry of murine exudates.

(c) Timeline: Mice were given zymosan (1 mg/ml, i.p.) two hours prior to inoculation of *E. coli* (10^5 CFU, i.p.). Peritoneal exudates were collected by lavaging at indicated time points. Total leukocytes were enumerated and leukocyte composition determined using flow cytometry.

(d) Representative flow cytometry of murine exudates.

Leukocyte (CD45+) subsets were determined using specific antibodies: Monocyte (CD45+ CD11b+ Ly6C+), PMN (CD45+ CD11b+ Ly6G+), Macrophage (CD45+ CD11b+ F4/80+).

Supplementary Fig. 2

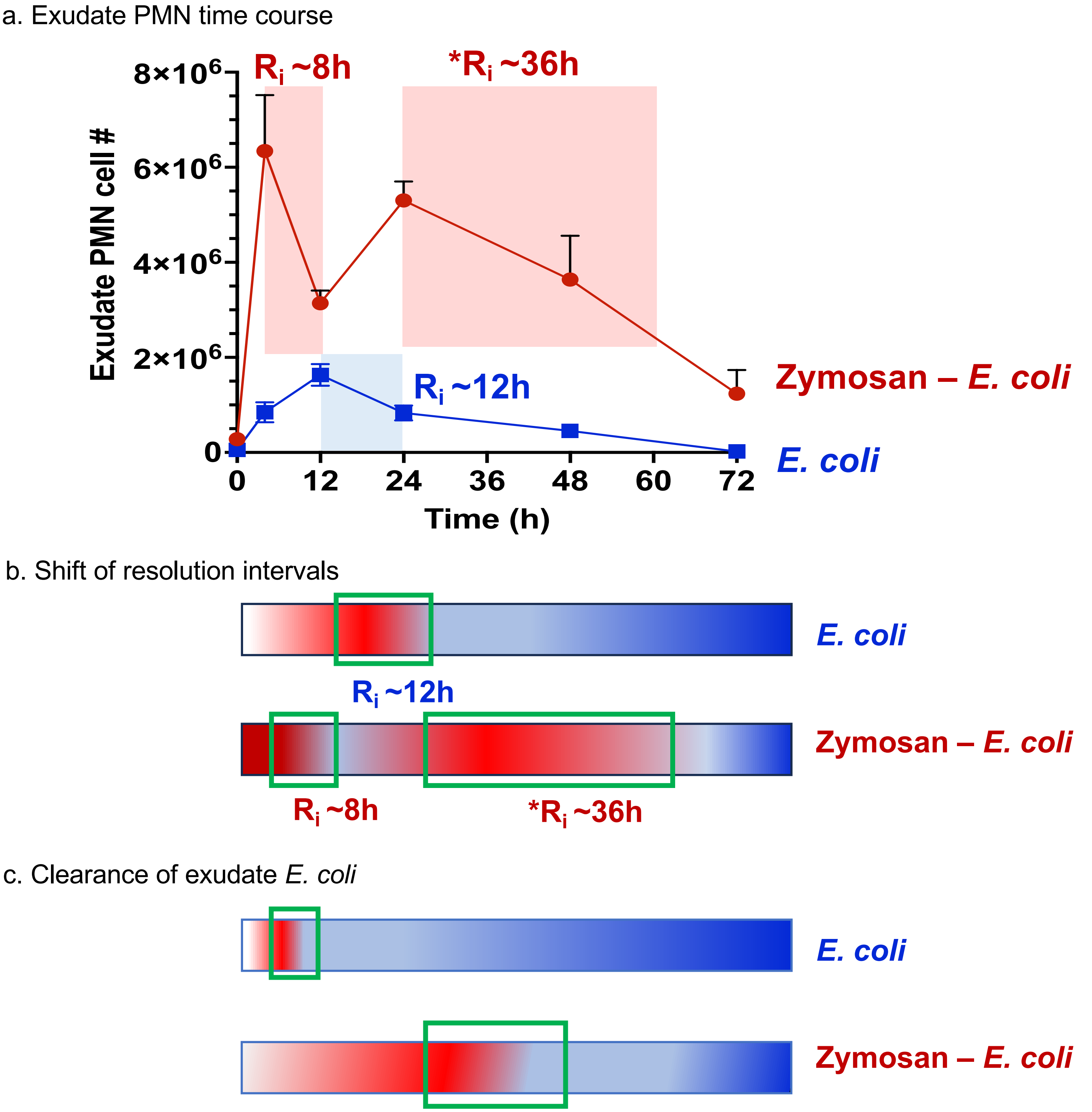

Supplementary Figure 2. Zymosan shifts inflammation-resolution clock of *E. coli* challenge.

(a) Exudates PMN time course during *E. coli* infection

(b) Shift of resolution intervals: comparison of *E. coli* alone versus ongoing inflammation with *E. coli* challenge

(a) Delay of exudate *E. coli* clearance by ongoing zymosan-initiated inflammation

PGE_2

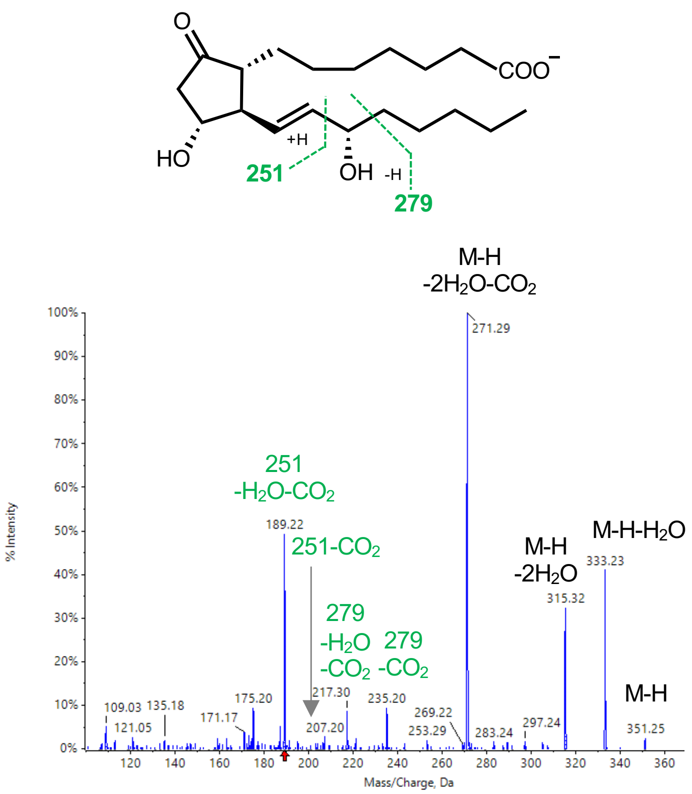

LTB_4

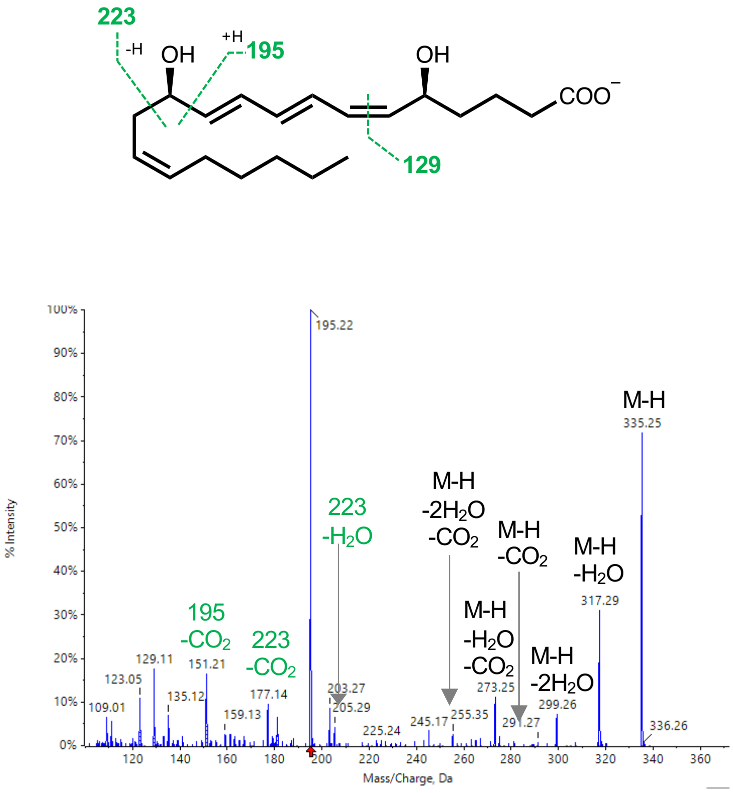

LXA_4

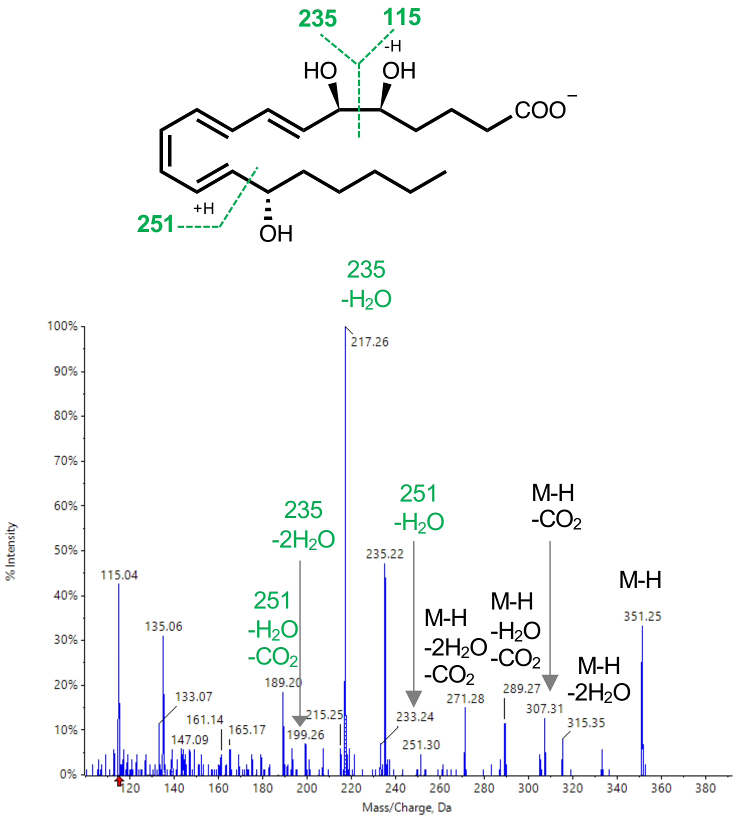

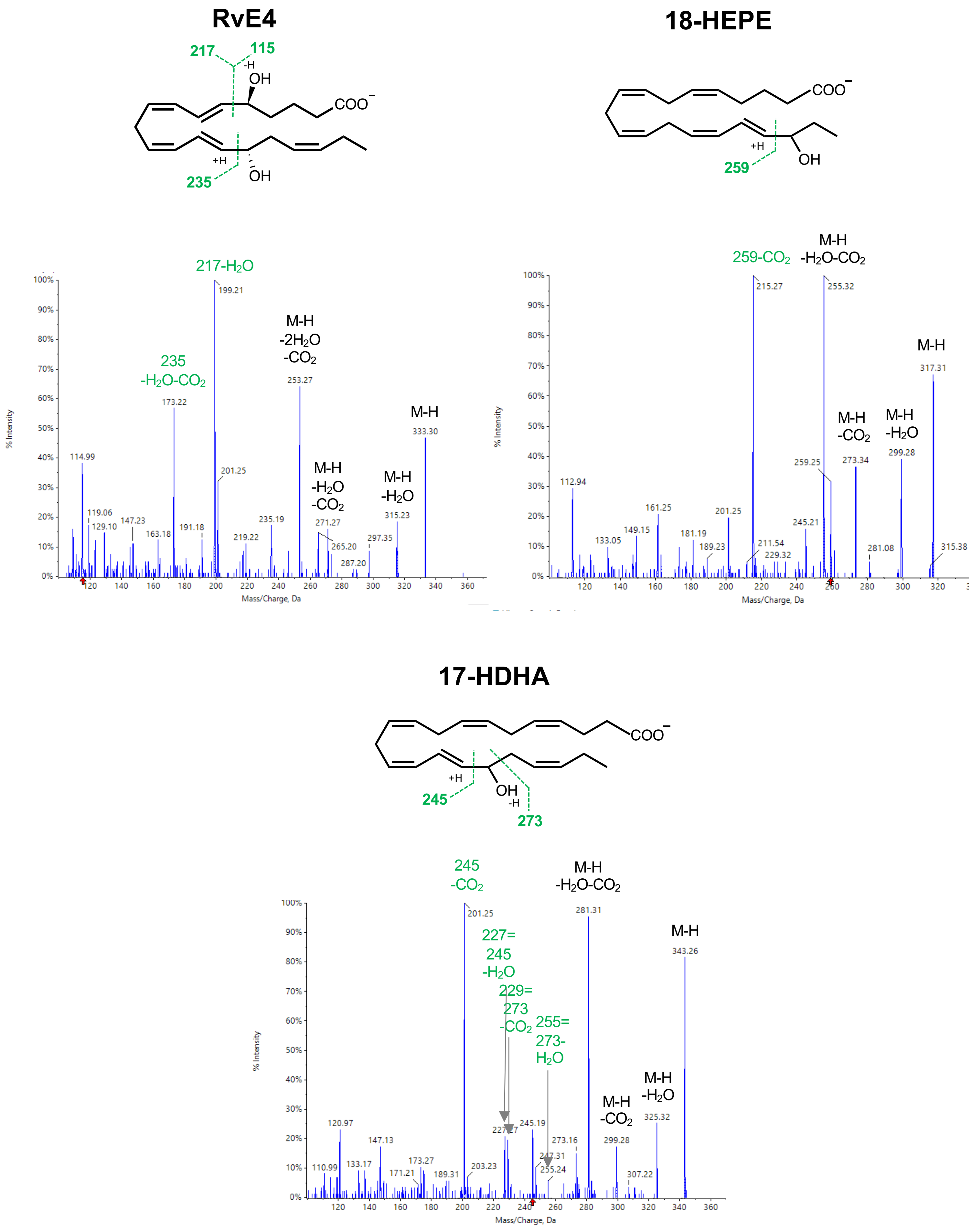
RvE4
217
115
-H
OH
COO−
+H
235
OH
18-HEPE
COO−
+H
259
OH
217-H2O
199.21
235
-H2O-CO2
173.22
M-H
-2H2O
-CO2
253.27
M-H
333.30
M-H
-H2O
-CO2
271.27
M-H
-H2O
315.23
114.99
119.06
129.10
147.23
163.18
191.18
201.25
219.22
235.19
265.20
287.20
297.35
% Intensity
Mass/Charge, Da
259-CO2
215.27
M-H
-H2O-CO2
255.32
M-H
317.31
M-H
-CO2
273.34
M-H
-H2O
299.28
259.25
112.94
133.05
149.15
161.25
181.19
189.23
201.25
211.54
229.32
245.21
281.08
315.38
17-HDHA
COO−
+H
245
OH
-H
273
245
-CO2
201.25
M-H
-H2O-CO2
281.31
M-H
343.26
227=
245
-H2O
229=
273
-CO2
255=
273-
H2O
M-H
-CO2
299.28
M-H
-H2O
325.32
120.97
110.99
133.17
147.13
171.21
173.27
189.31
203.23
245.19
247.31
255.24
273.16
307.22

Supplementary Fig. 3

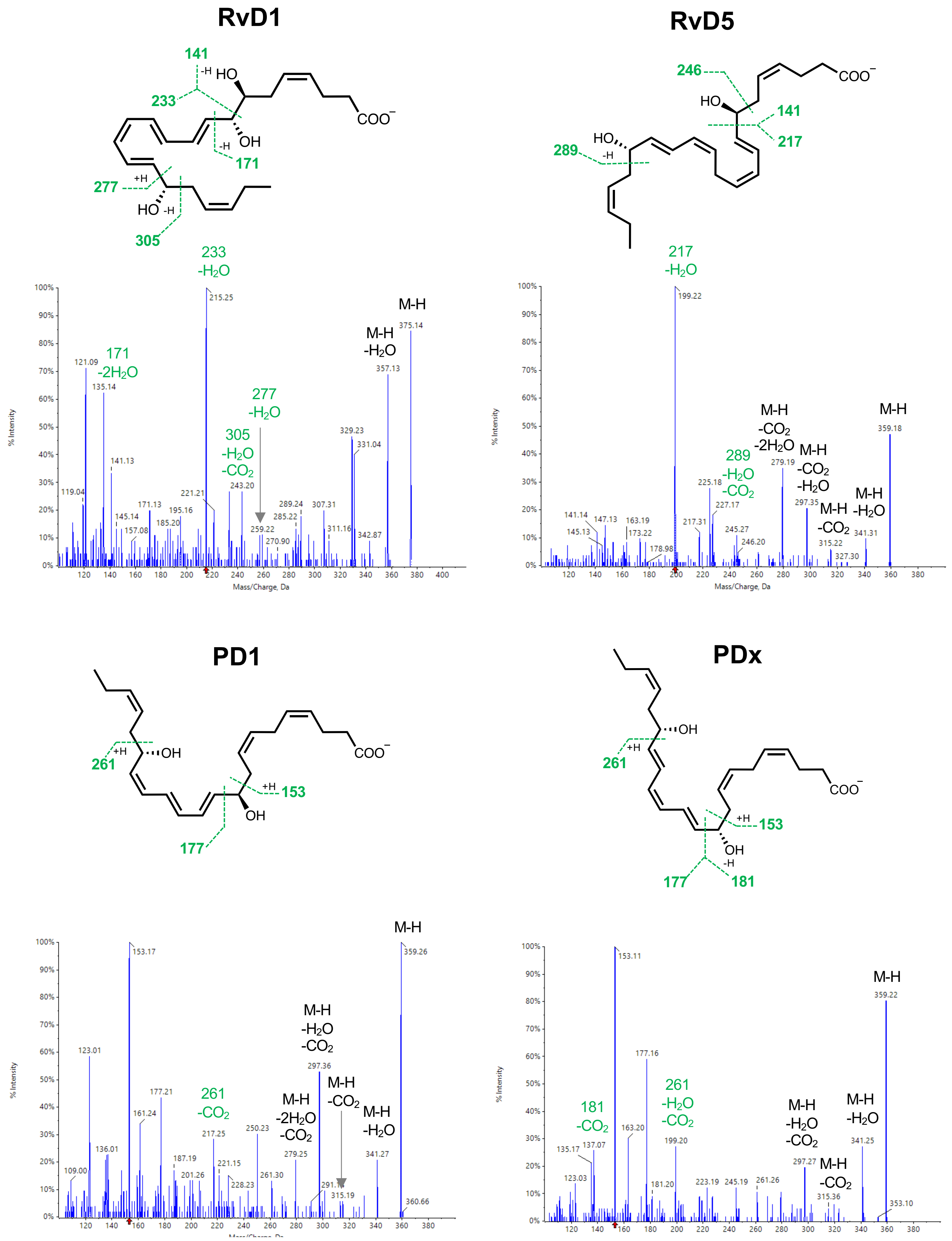

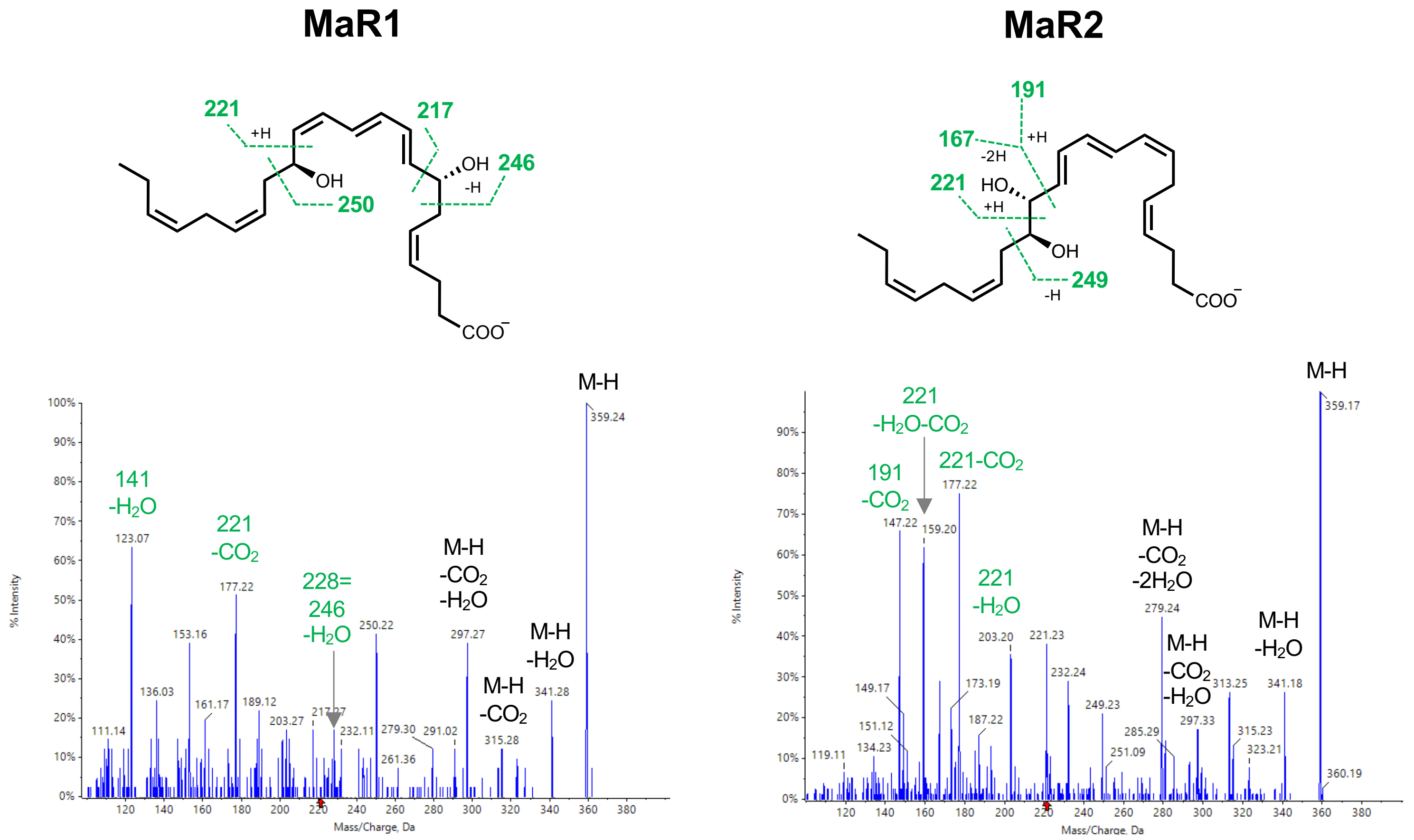

Supplementary Figure S3. MS-MS of synthetic LMs with structures and fragmentations. Ions obtained from neutral loss are in indicated in black on the MS-MS, and ions obtained from C-C backbone fragmentations are indicated in green on the MS-MS and structures. These MS-MS spectra are the enlarged versions of those shown in Fig. 2.

Supplementary Fig. 4

a. PLS-DA score plot and ANOVA: exudate SPMs and eicosanoids

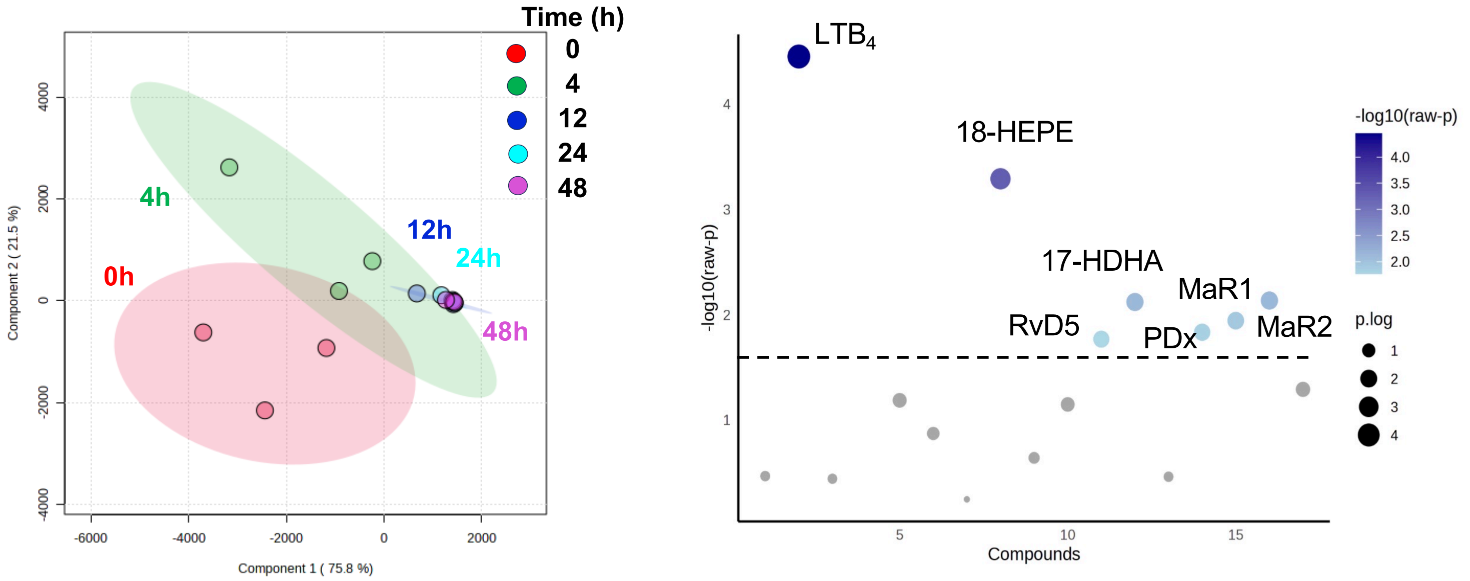

b. Hierarchical Clustering Heatmaps: exudate SPMs and eicosanoids

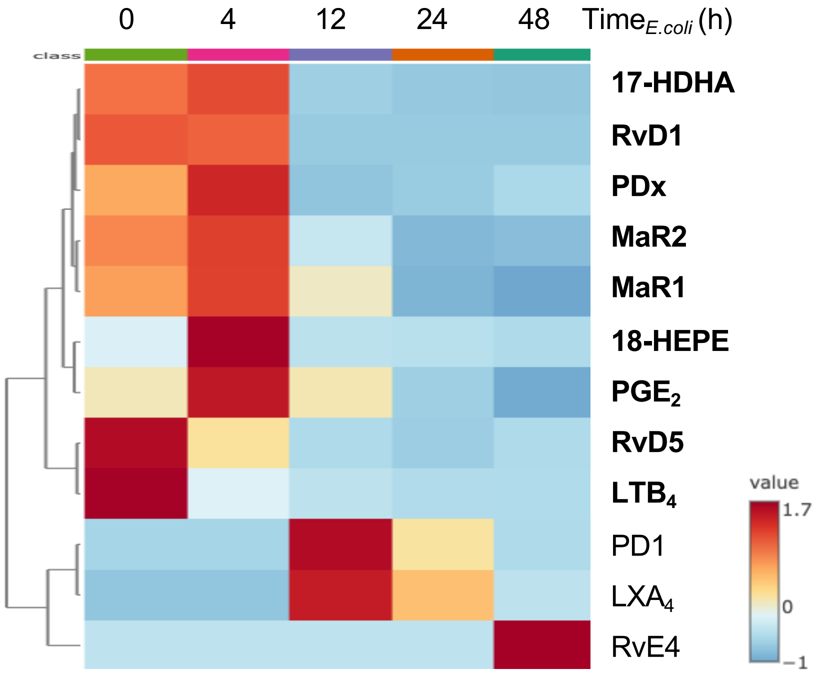

Supplementary Figure S4. SPM and eicosanoid signature profiles: *E. coli* challenge with ongoing zymosan-induced inflammation

(a) (left) PLS-DA score plot with each dot representing profiles from each mouse sample, and (right) ANOVA for identified LMs, SPMs and pathway markers in the exudates. ANOVA shows that 7 out of 12 identified SPMs and eicosanoids give significant temporal changes using Tukey's post-hoc test (P<0.05) during the time course 0-48h.

(b) A hierarchical Clustering Heatmap was generated using normalized data with autoscale features. Euclidean distance was used for distance measure and Ward's method was applied in hierarchical cluster analysis. Averages of n=3 for each SPM and eicosanoid in each time point are shown.

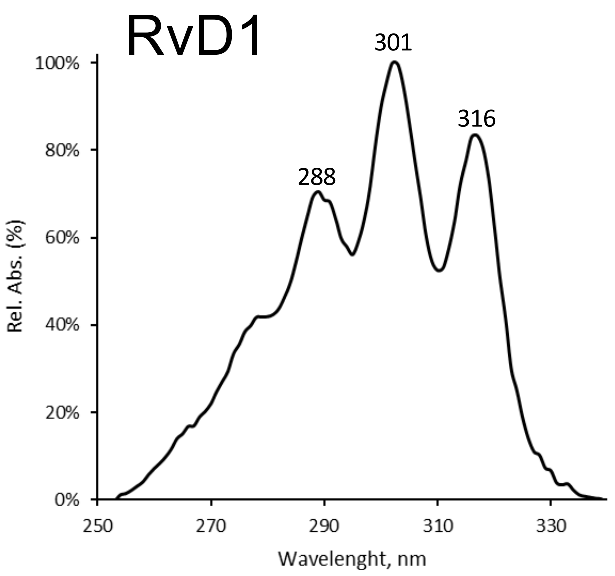
RvD1
100%
80%
60%
40%
20%
0%
Rel. Abs. (%)
301
316
288
250
270
290
310
330
Wavelenght, nm

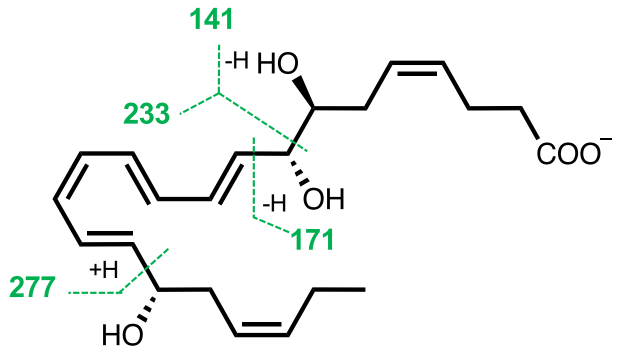
141
-H HO
233
COO⁻
-H OH
171
+H
277
HO

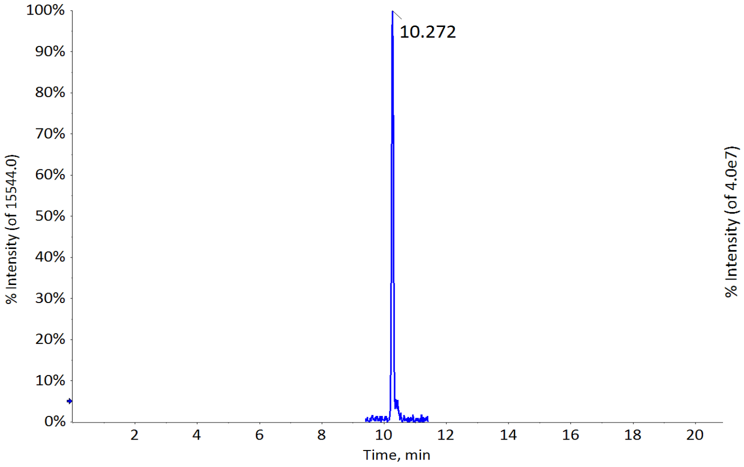
100%
90%
80%
70%
60%
50%
40%
30%
20%
10%
0%
% Intensity (of 15544.0)
10.272
2
4
6
8
10
12
14
16
18
20
Time, min

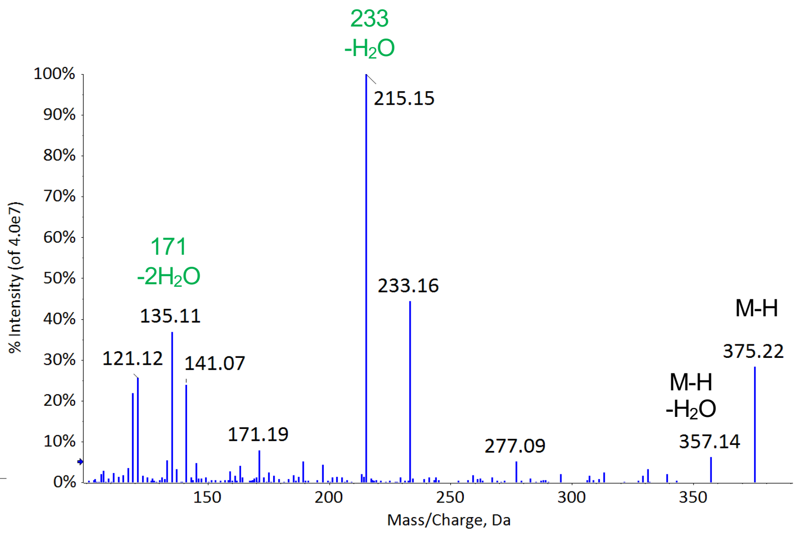
233
-H2O
215.15
100%
90%
80%
70%
60%
50%
40%
30%
20%
10%
0%
% Intensity (of 4.0e7)
171
-2H2O
135.11
121.12
141.07
171.19
233.16
277.09
M-H
375.22
M-H
-H2O
357.14
150
200
250
300
350
Mass/Charge, Da

Supplementary Fig. 5b

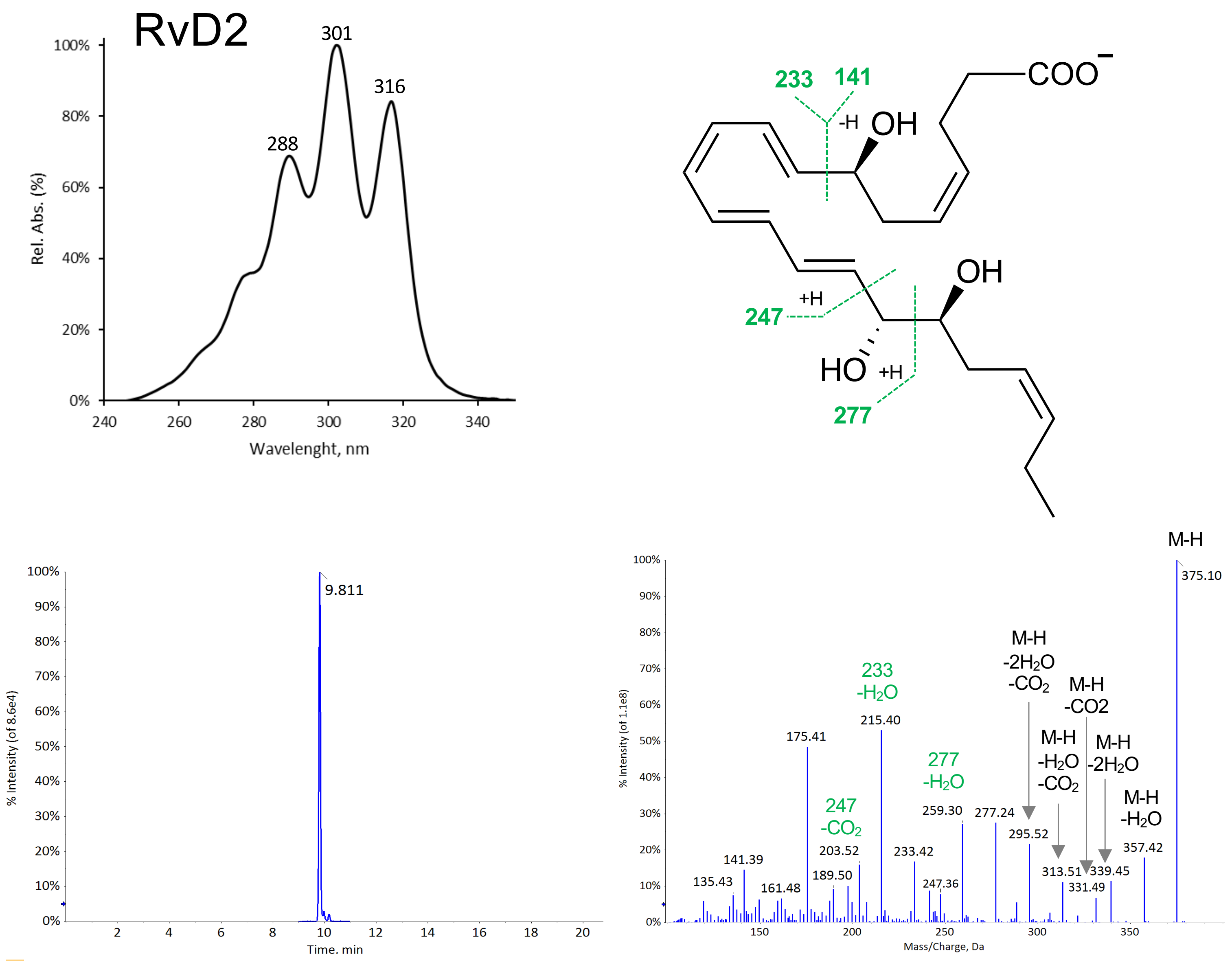

RvD5

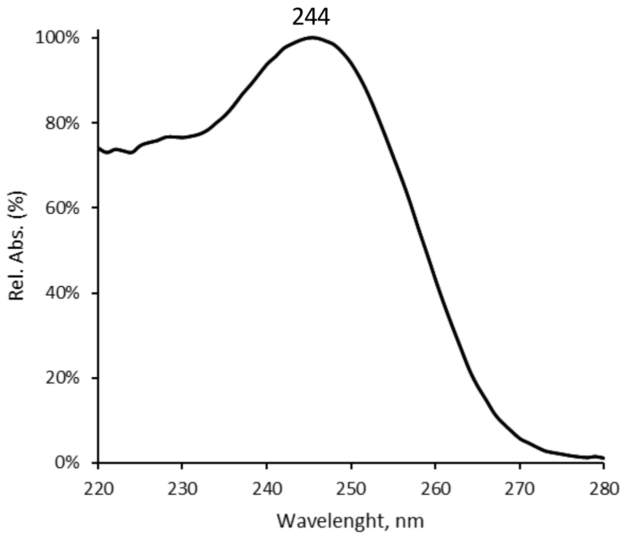
244
100%
80%
60%
40%
20%
0%
Rel. Abs. (%)
220
230
240
250
260
270
280
Wavelenght, nm

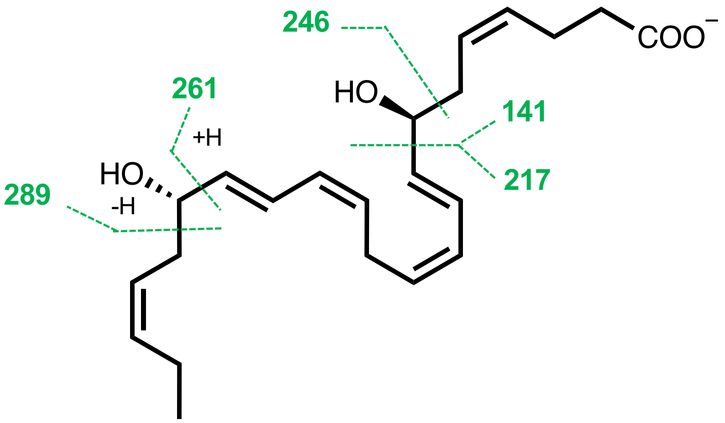
246
COO–
HO
261
+H
141
217
HO
-H
289

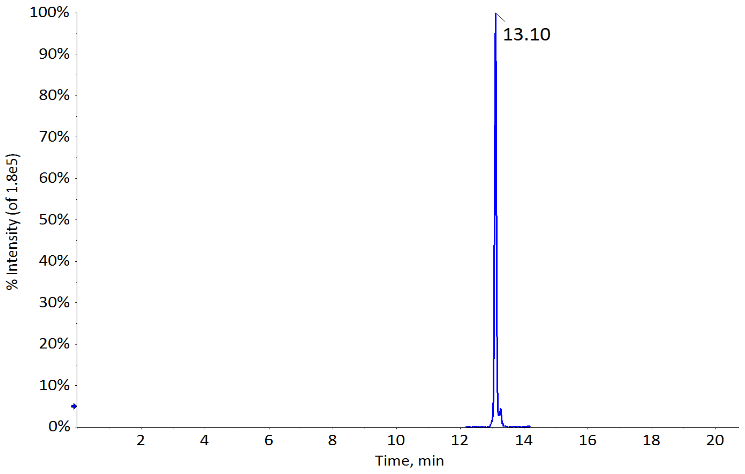
100%
90%
80%
70%
60%
50%
40%
30%
20%
10%
0%
% Intensity (of 1.8e5)
13.10
2
4
6
8
10
12
14
16
18
20
Time, min

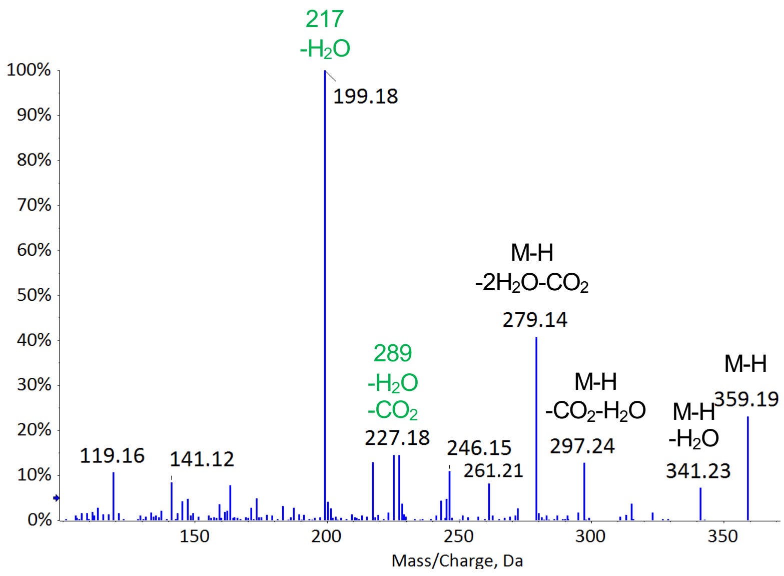
217
-H2O
199.18
100%
90%
80%
70%
60%
50%
40%
30%
20%
10%
0%
M-H
-2H2O-CO2
279.14
289
-H2O
-CO2
227.18
M-H
-CO2-H2O
297.24
M-H
359.19
M-H
-H2O
341.23
119.16
141.12
246.15
261.21
150
200
250
300
350
Mass/Charge, Da

Supplementary Fig. 5d

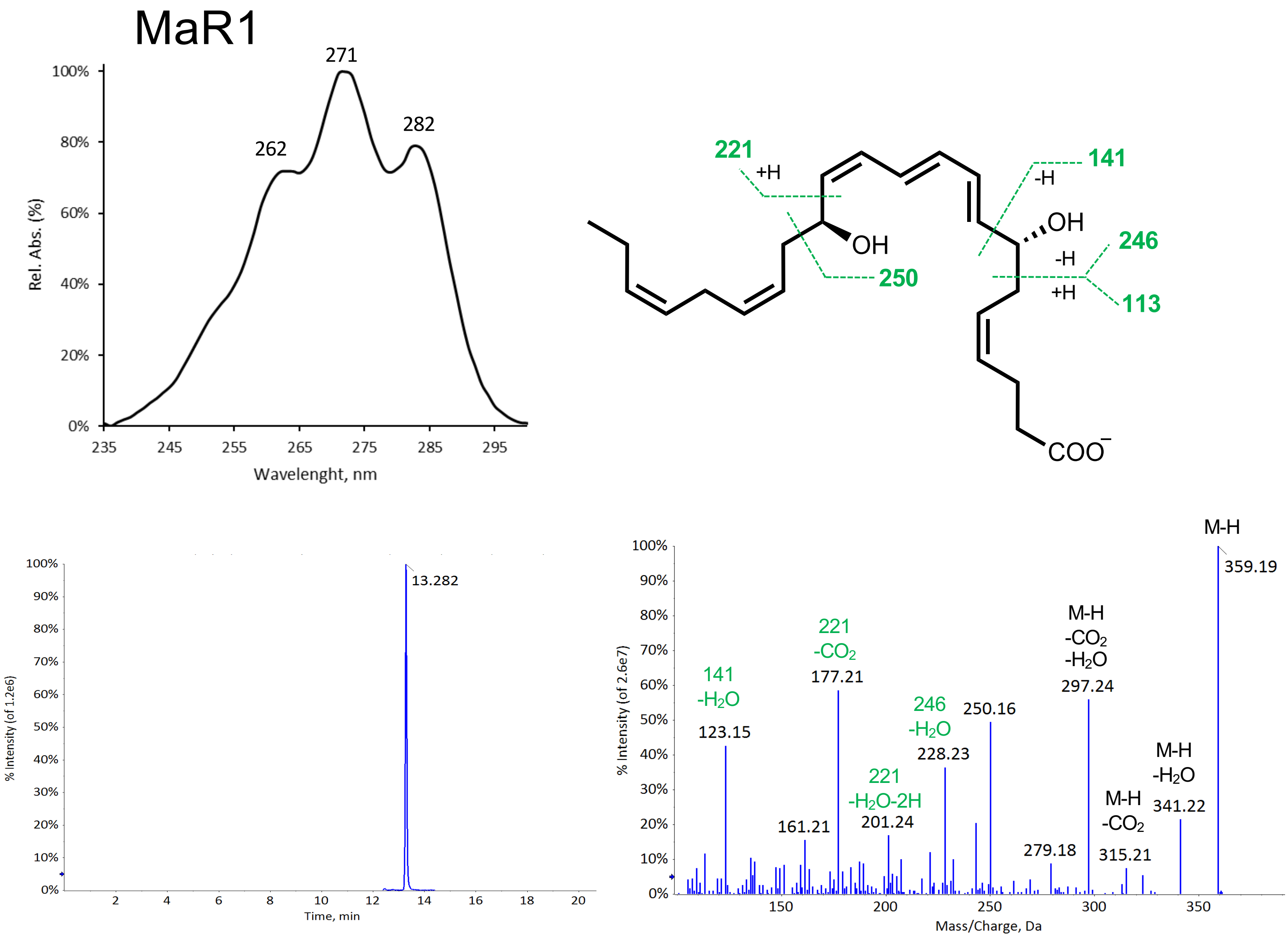

Supplementary Fig. 5e

RvE2

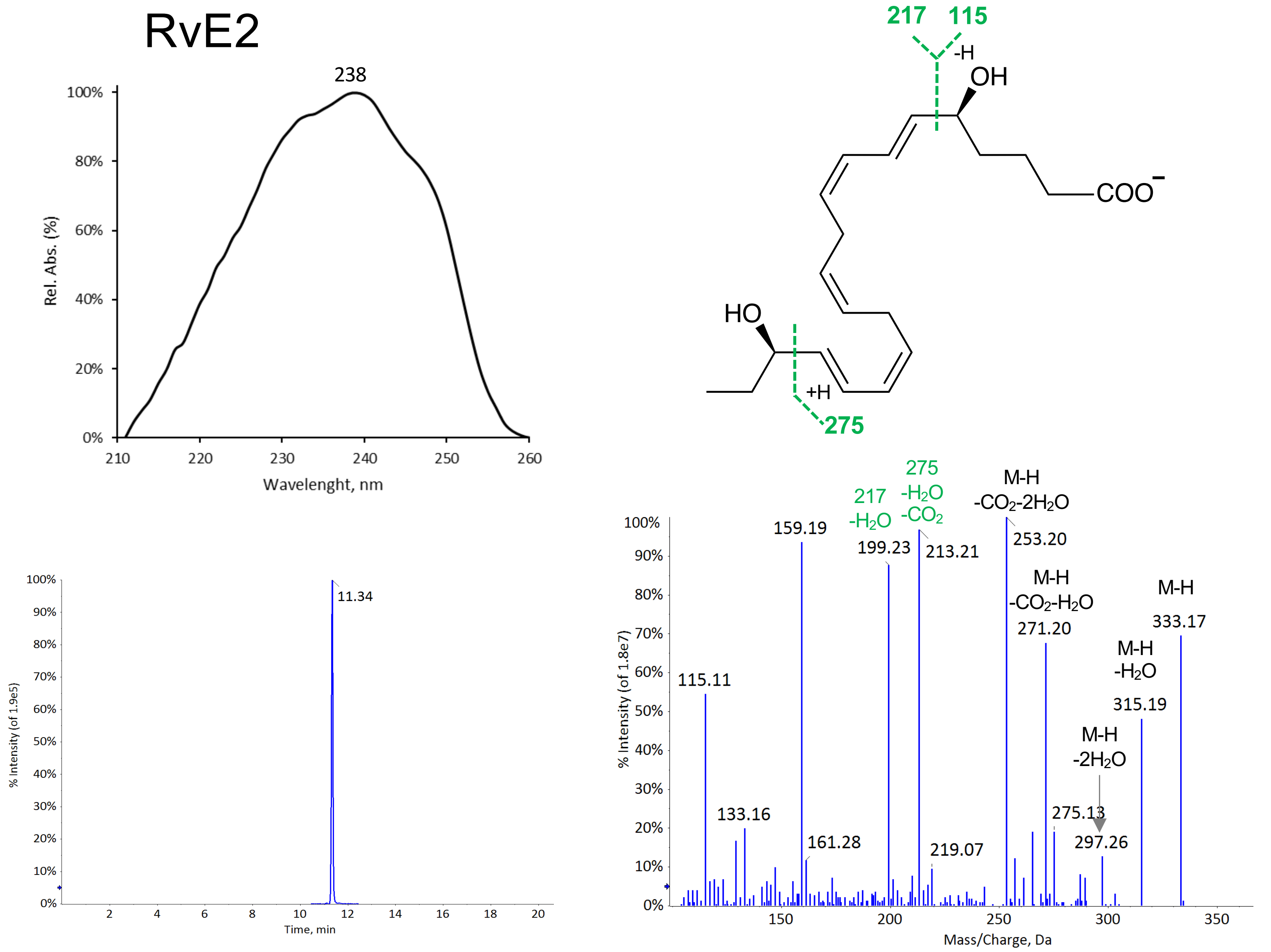

Supplementary Figure S5. Authentication of RvD1, RvD2, RvD5, MaR1 and RvE2, and their proposed MS/MS fragmentation.

The integrity of synthetic (a) RvD1, (b) RvD2, (c) RvD5, (d) MaR1 and (e) RvE2 used for *in vivo* experiments were each assessed using UV spectroscopy and liquid chromatography/tandem mass spectrometry (LC-MS/MS) to obtain UV spectra, retention times (T_R, min) and diagnostic MS/MS spectra. For each compound, Top left: UV spectrum showing maximum absorbance. (Bottom left) MRM chromatogram with a specific retention time. (Top right) Chemical structure and proposed MS/MS fragmentations and (Bottom right) tandem MS/MS; ions obtained from neutral loss are in indicated in black on the MS-MS, and ions obtained from C-C backbone fragmentations are indicated in green on the MS-MS and structures.

(a) RvD1: UV spectrum: RvD1 possesses a conjugated tetraene chromophore, that gave a UV absorption spectrum with λ^{MeOH}_{max} ≃301 nm and shoulders ≃288 and 316 nm. MRM chromatogram: MRM of *m/z* 375>215 and T_R 10.27 min. MS-MS ions: *m/z* 375=M-H, 357=M-H-H_2O, 277=M-$CHOHCH_2(CH)_2CH_2CH_3$, 233=M-H-$CHOHCH_2(CH)_2(CH_2)_2CO_2$, 215= 233-$H_2O$, 171=M-H-$(CH)_8CHOHCH_2(CH)_2CH_2CH_3$-H, 141=M-H-$CHOH(CH)_8CHOHCH_2(CH)_2CH_2CH_3$,135=171-2$H_2O$.

(b) RvD2: UV spectrum: RvD2 possesses a conjugated tetraene chromophore, that gave a UV with λ^{MeOH}_{max} ≃301 nm and shoulders ≃288 and 316 nm. MRM chromatogram: MRM of *m/z* 375>141 and T_R 9.81 min. MS-MS ions: *m/z* 375=M-H, 357=M-H-H_2O, 339=M-H-2H_2O, 331=M-H-CO_2, 313=M-H-H_2O-CO_2, 295=M-H-2H_2O-CO_2, 277=M-$CHOHCH_2(CH)_2CH_2CH_3$, 259=277-H_2O, 247=M-$(CHOH)_2CH_2(CH)_2CH_2CH_3$, 241=277-2$H_2O$, 233=M-H-$CHOHCH_2(CH)_2(CH_2)_2CO_2$, 215=233-$H_2O$, 203=247-$CO_2$, 175=M-H-[$CH_2(CH)_2(CH_2)_2CO_2$]-[$CH_2(CH)_2CH_2CH_3$]-$H_2O$-2H, 141=M-H-$CHOH(CH)_8(CHOH)_2CH_2(CH)_2CH_2CH_3$-H.

(c) RvD5: UV spectrum: RvD5 possesses two conjugated diene chromophores, that gave a UV with λ^{MeOH}_{max} ≃244 nm. MRM chromatogram: MRM of *m/z* 359>199 and T_R 13.10 min. MS-MS ions: *m/z* 359=M-H, 341=M-H-H_2O, 297=M-H-H_2O-CO_2, 279=M-H-2H_2O-CO_2, 261=M-$CHOHCH_2(CH)_2CH_2CH_3$, 246=M-H-$CH_2(CH)_2(CH_2)_2CO_2$, 227=289-H_2O-CO_2, 217=$(CH)_4CH_2(CH)_4CHOHCH_2(CH)_2CH_2CH_3$, 199=217-$H_2O$, 141=$CHOHCH_2(CH_2)_2CO_2$

(d) MaR1: UV spectrum: MaR1 possesses a conjugated triene, that gave a UV with λ^{MeOH}_{max} ≃271 nm and shoulders ≃262 and 282 nm. MRM chromatogram: MRM of *m/z* 359>177 and T_R 13.28 min. MS-MS ions: *m/z* 359=M-H, 341=M-H-H_2O, 315=M-H-CO_2, 297=M-H-H_2O-CO_2, 250=M-H-$CH_2(CH)_2CH_2(CH)_2CH_2CH_3$, 228=246-$H_2O$, 221=M-$CHOHCH_2(CH)_2CH_2(CH)_2CH_2$-$CH_3$, 201=221-$H_2O$-2H, 177=221-$CO_2$,123=141-$H_2O$, 113=M-$CHOH(CH)_6CHOHCH_2(CH)_2CH_2(CH)_2CH_2CH_3$.

(e) RvE2: UV spectrum: RvE2 possesses two conjugated dienes, that gave a UV with λ^{MeOH}_{max} ≃238 nm. MRM chromatogram: MRM of *m/z* 333>215 and T_R 11.34 min. MS-MS ions: *m/z* 333=M-H, 315=M-H-H_2O, 297=M-H-2H_2O, 271=M-H-H_2O-CO_2, 253=M-H-2H_2O-CO_2, 275=M-$CHOHCH_2CH_3$, 213=275-H_2O-CO_2, 199=217-H_2O, 159=M-H-[$CHOHCH_2CH_3$]-[$CHOH(CH_2)_3CO_2$], 115=M-H-$(CH)_4CH_2(CH)_2CH_2(CH)_4CHOHCH_2CH_3$-H.

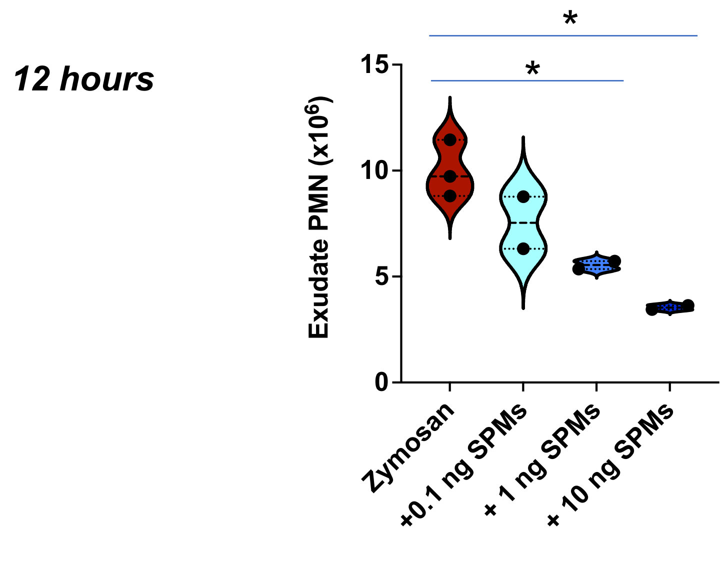

Supplementary Figure S6. A SPM panel in combination regulates exudate PMNs: dose response

Mice were administered zymosan (1 mg/mL) together with vehicle or a panel of SPMs in combination (RvD1, RvD2, RvD5, MaR1 and RvE2, i.p.) at 0.1, 1 or 10 ng each per mouse. At 12 h, peritoneal exudates were collected, total leukocytes were enumerated and PMNs determined by flow cytometry. *P<0.05, vs zymosan alone using one-way ANOVA with Tukey's multiple comparison test.

Supplementary Fig. 7

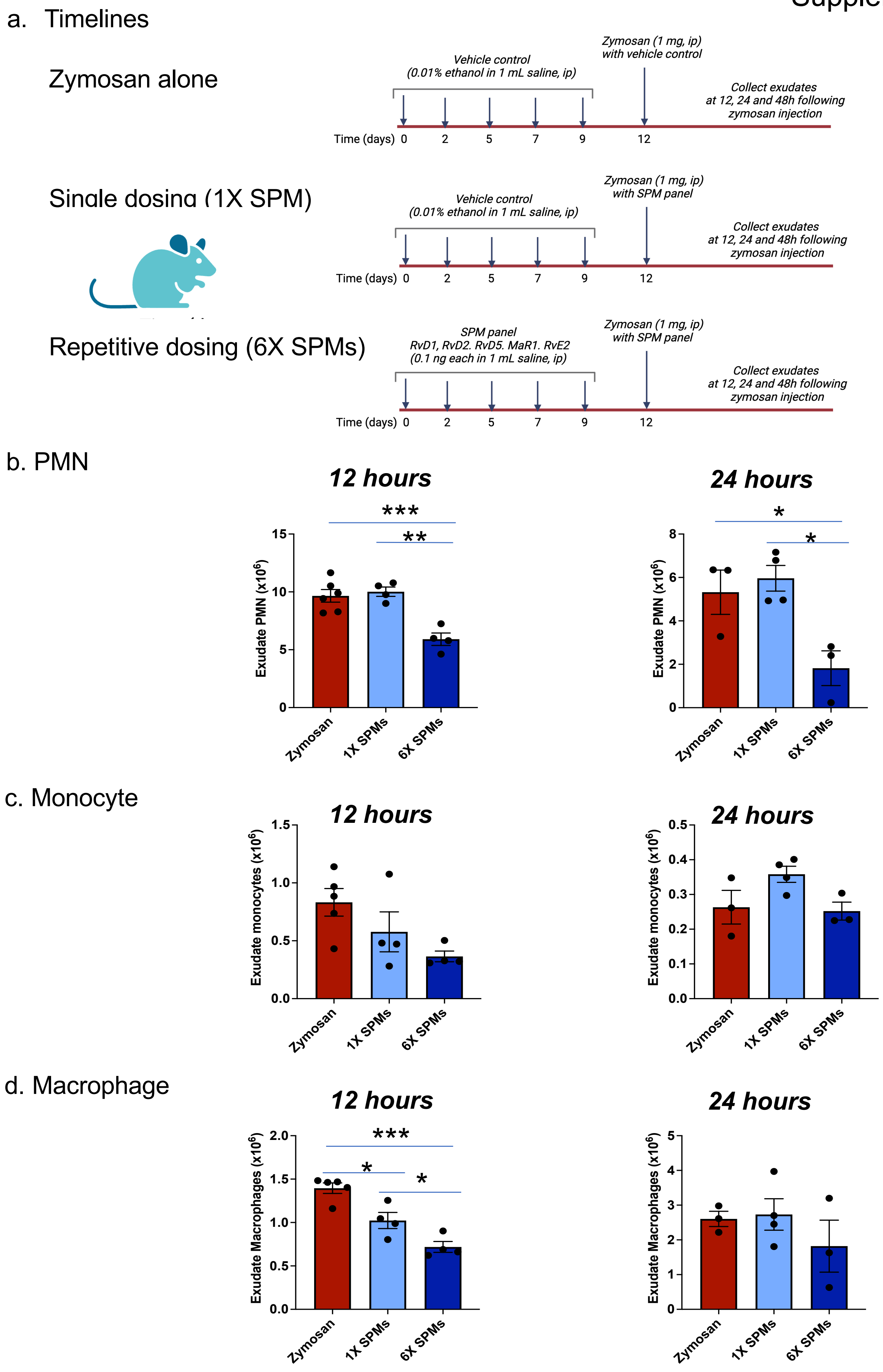

Supplementary Figure S7. SPM training *in vivo: exudate* PMN, monocyte and macrophage numbers

(a) Timelines:

single dosing (1X SPMs) -- Mice were administered with vehicle (0.01% ethanol in 1 mL saline, i.p.) 5 times on Day 0, 2, 5, 7 and 9. Then on day 12, mice were given the SPM panel together with zymosan A (1 mg/mouse, i.p.) to initiate peritonitis.

Repetitive dosing (6X SPMs) -- Mice were administered with a panel of SPMs (RvD1, RvD2, RvD5, MaR1 and RvE2, 0.1 ng/mouse each SPM, i.p.) 5 times on Day 0, 2, 5, 7 and 9. Then on day 12, mice were given the SPM panel together with zymosan A (1 mg/mouse, i.p.) to initiate peritonitis.

(b-d) Exudates were collected at indicated time points. Total leukocytes were enumerated and cell compositions determined by flow cytometry (see Fig. 5 for gating strategy). Time course of (b) PMN (c) monocyte and (d) macrophage numbers. *$P<0.05$, **$P<0.01$, ***$P<0.001$ using one-way ANOVA with Tukey's multiple comparison test.

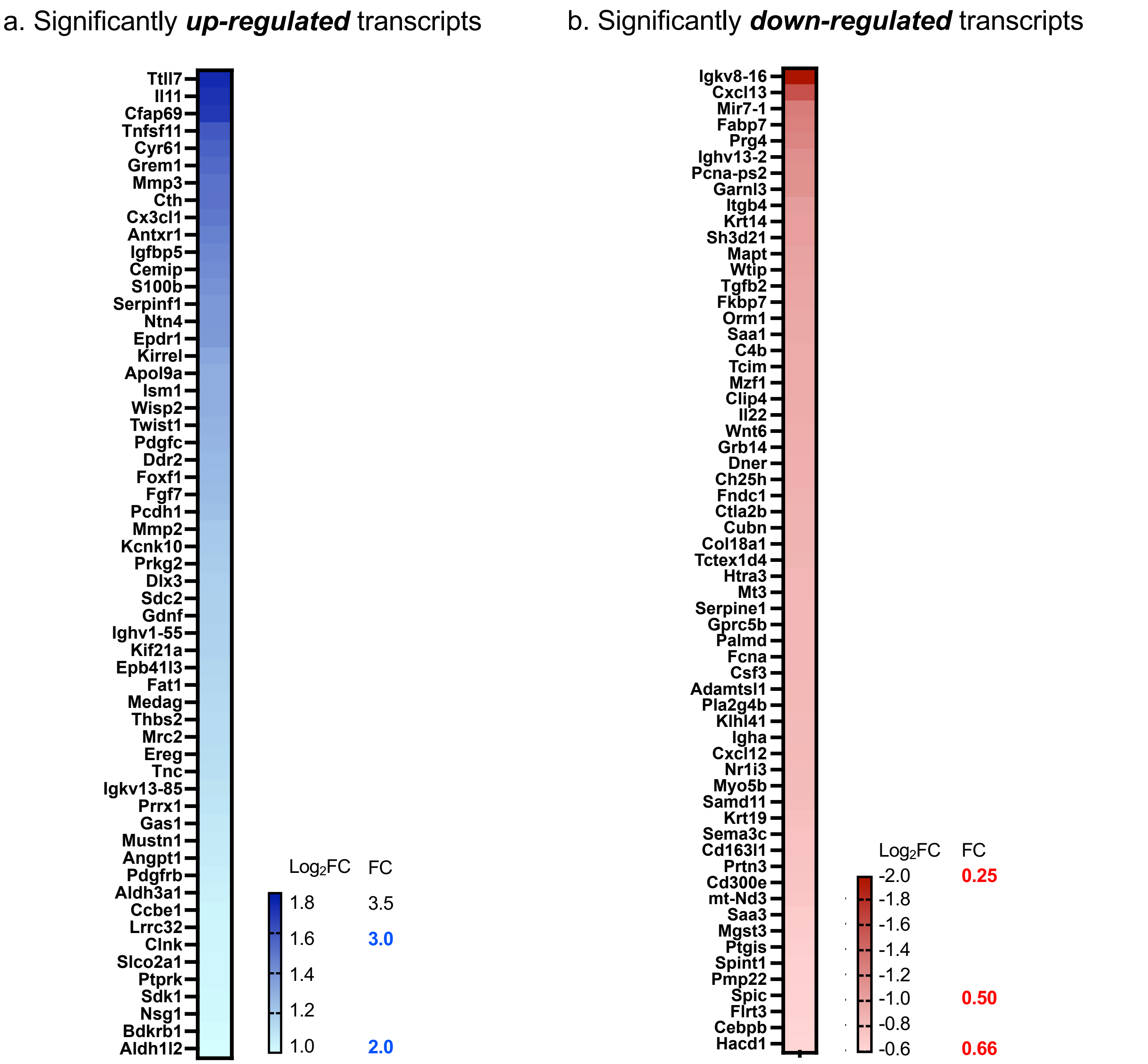

Supplemental Figure S8. RNA-seq transcript analysis.

Mice were administered with vehicle or a panel of SPMs (RvD1, RvD2, RvD5, MaR1 and RvE2, 0.1 ng each per mouse, i.p.) 5 times on Day 0, 2, 5, 7 and 9. On day 12, peritonitis was initiated by zymosan A (1 mg/mouse, i.p.), together with vehicle or SPM panel, and exudates collected at 12h for RNA-seq (n=4 mouse exudates in each group). RNA-seq transcript analysis demonstrates that SPM repetitive dosing significantly (a) up-regulated transcripts with $Log_2FC>1$ and (b) down-regulated transcripts with $Log_2FC<-0.6$, by subthreshold SPMs plus zymosan compared to zymosan alone.

a. Inflammation-resolution pathway analysis

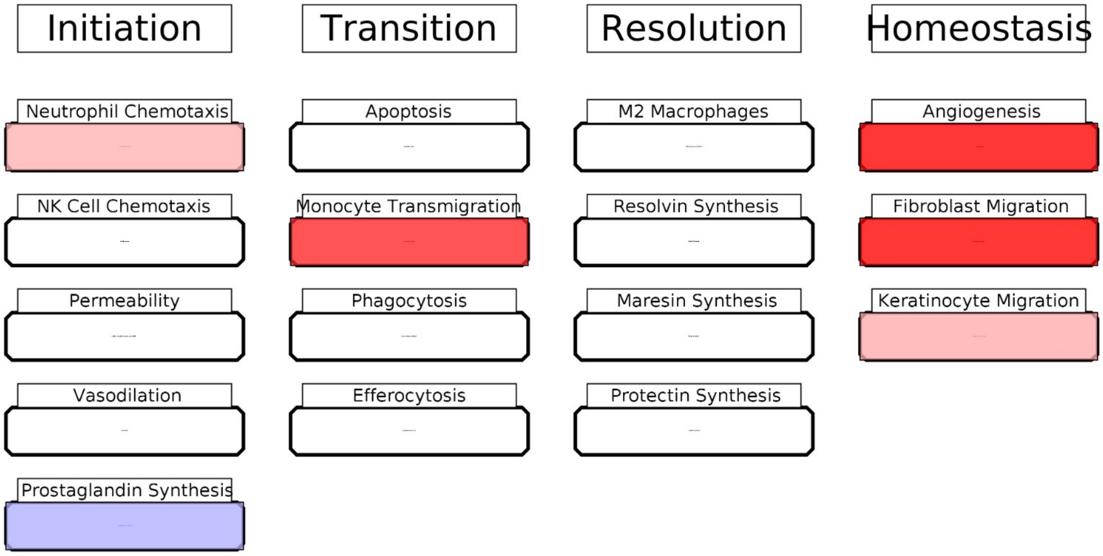

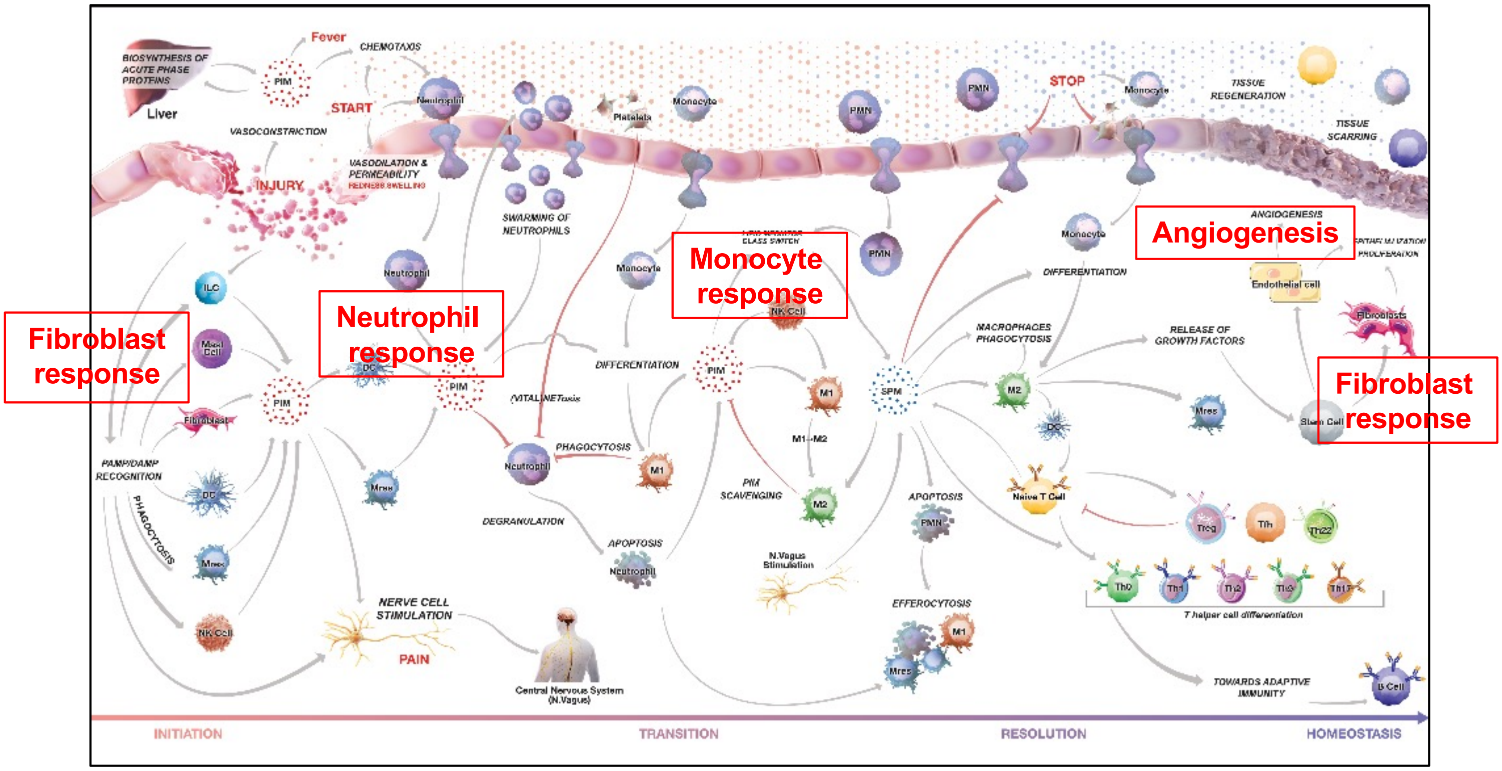

Phenotype	log_2FC	log_2FC_pvalue	Genes
angiogenesis	1.2	0.000907	PDGFRB, ANGPT1, ANGPTL4, MMP2, BLVRB
fibroblast response	1.03	6.55E-06	FGF7, PDGFRB, EDN1
monocyte response	0.85	0.001433	TWIST1, VCAM1, MMP2, RAC1, TLR4, MMP14
myofibroblast response	0.43	0.030712	FGF7, PDGFRB, EDN1
keratinocyte response	0.33	0.000319	FGF7, EDN1, PDGFRB, IGF1R
neutrophil response	0.3	0.000102	CXCR2, MIF

b. Central Regulatory Network (CRN)

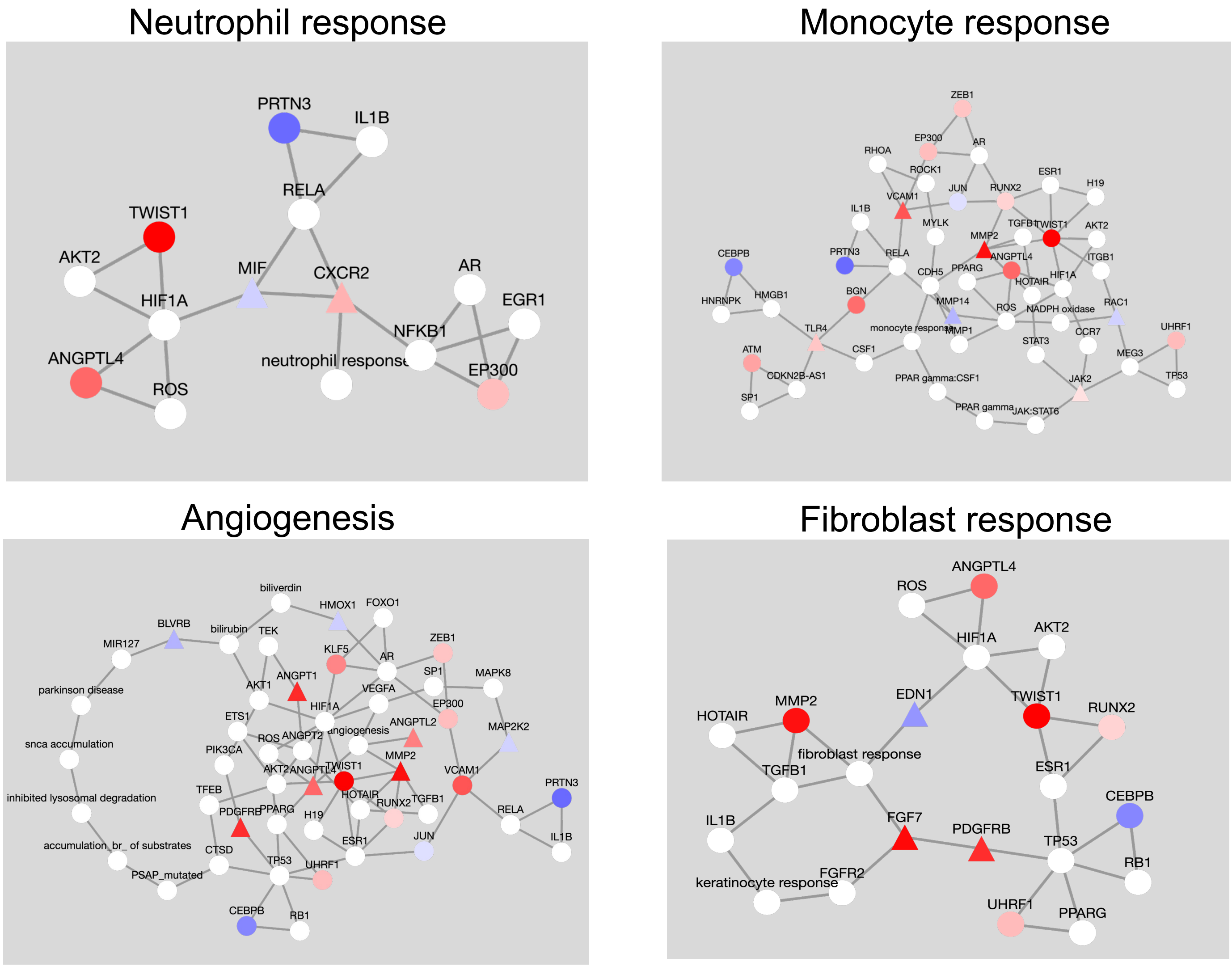

Supplemental Figure S9. Network analyses using Atlas of Inflammation Resolution (AIR)

(a) (top) Inflammation-resolution processes and phenotypes were grouped into 4 phases (inflammation initiation, transition, resolution, and homeostasis). The processes and phenotype significantly regulated by SPMs were normalized between + 1 (upregulation; red color) and -1 (downregulation; blue color). (Middle) Repetitive subthreshold SPMs up-regulated select phenotypes in each phase, highlighted in red. (bottom) List of genes that were significantly up-regulated by SPMs in these pathway.

(b) Network- and expression-based motif ranking creates a central regulatory network (CRN) representing the molecular interaction associated with the selected phenotype element (e.g., monocyte response, fibroblast response). The CRN highlights the up-regulated (red) or down-regulated (blue) genes (padj ≤ 0.05) by SPMs.

Table S1. LC conditions and MS acquisition parameters used on SCIEX QTRAP 6500+ and Triple Quad 7500 systems‡

	**SCIEX QTRAP 6500+ LowMass**		**SCIEX Triple Quad™ 7500 LowMass**	
**LC system**	Shimadzu LC-20AD		SCIEX ExionLC	
Column	Phenomenex Kinetex® 2.6 µm Polar C18 100Å (100 x 3 mm)		Phenomenex Kinetex® 2.6 µm PS C18 100Å (100 x 3 mm)	
Solvent A	Water (0.1% formic acid)		Water (0.1% formic acid)	
Solvent B	Methanol (0.1% formic acid)		Methanol (0.1% formic acid)	
Flow Rate	0.5 mL/ min		0.5 mL/ min	
Gradient	Time (min)	Solvent B (%)	Time (min)	Solvent B (%)
	0.0	Start	0.0	Start
	0.1	45	0.1	45
	2.0	45	2.0	45
	16.5	80	16.5	80
	16.6	98	16.6	98
	18.5	98	18.5	98
	18.6	10	18.6	10
	20.9	10	20.9	10
**Source and Gas Parameters**				
Ion Source	IonDrive™ Turbo V		OptiFlow® Pro	
Curtain Gas	30		40	
CAD Gas	12		12	
Temperature (°C)	520		500	
Ion Source Gas 1 (psi)	85		45	
Ion Source Gas 2 (psi)	50		70	
**EPI and IDA Criteria**				
Scan Type	MRM		MRM	
Polarity	Negative		Negative	
Spray Voltage (V)	-4200		-2000	
Q1; Q3 resolution	Unit		Unit	

‡LC-ESI-MS/MS, liquid chromatography coupled with electrospray ionization tandem mass spectrometry; EPI, enhanced product ion scanning; IDA, information-dependent acquisition; MRM, multiple reaction monitoring.

Table S2. Quantification of lipid mediators and pathway markers in murine *E. coli* infectious exudates‡

Time E. coli	0h			4h			12h			24h			48h		
**AA-metabolome**															
**PGE_2**	58.2	±	15.4	451.7	±	402.6	44.1	±	10.7	32.4	±	8.4	42.5	±	22.3
**LTB_4**	55.8	±	29.9	238.1	±	193.9	51.7	±	28.6	14.9	±	3.8	42.2	±	37.7
**LXA_4**		*			*		15.2	±	4.2	7.9	±	5.0	11.3	±	4.6
**EPA-metabolome**															
**RvE4**		*			*			*			*		8.8	±	8.4
**18-HEPE**	112.8	±	53.3	475.9	±	162.9	18.4	±	8.1	15.9	±	6.6	26.7	±	8.1
**DHA-metabolome**															
**RvD1**		*			*			*			*			*	
**RvD5**	27.1	±	13.6	42.6	±	18.3		*			*		6.3	±	5.7
**PD1**	3.4	±	3.4		*		21.2	±	10.2	16.2	±	5.8	63.4	±	35.3
**PDx**	76.8	±	42.1	154.4	±	113.2	13.5	±	4.8	20.8	±	10.2	53.2	±	45.9
**17-HDHA**	1640.4	±	372.7	2410.4	±	1023.5	153.5	±	36.2	243.6	±	92.6	747.6	±	327.9
**MaR1**	350.7	±	217.0	150.9	±	58.1	107.8	±	44.1	89.3	±	9.7	474.0	±	205.3
**MaR2**	42.0	±	12.3	74.2	±	59.5		*			*		2.8	±	2.8

‡Quantification of lipid mediators and their pathway markers in infectious exudates at time 0, 4h, 12h, 24h and 48h following *E. coli* challenge. Results are expressed as mean±SEM in picograms (in 2 mL mouse exudate) from three separate experiment, calculated using GraphPad Prism (Grouped analyses - Row statistics). For each time point in each experiment, exudates from 4 mice were pooled for LM extraction and LC-MS-MS carried out using a Sciex QTRAP 6500+. * denotes lipid mediator that does not meet the criteria for identification. These criteria are: MS/MS library fit score >70 with a signal-to-noise ratio > 5 (see Methods).

Table S3. Quantification of lipid mediators and pathway markers in murine infectious exudates with zymosan-induced inflammation followed by *E. coli*‡

Time $_{E. coli}$	0h			4h			12h			24h			48h		
**AA-metabolome**															
**PGE$_2$**	373.5	±	61.7	857.8	±	537.7	380.7	±	299.3	146.4	±	57.3	43.2	±	19.8
**LTB$_4$**	2856.8	±	473.0	435.1	±	260.0	132.8	±	94.5	22.6	±	3.9	21.3	±	18.7
**LXA$_4$**		*			*		31.8	±	26.1	16.9	±	8.9	4.7	±	1.4
**EPA-metabolome**															
**RvE4**		*			*			*			*		3.3	±	3.3
**18-HEPE**	114.3	±	32.1	717.8	±	176.8	42.4	±	32.2	32.4	±	25.2	13.9	±	4.8
**DHA-metabolome**															
**RvD1**	12.7	±	6.6	12.2	±	9.3		*			*			*	
**RvD5**	172.7	±	65.8	64.3	±	16.0	14.2	±	9.8	4.9	±	1.9	14.1	±	13.9
**PD1**		*			*		35.7	±	28.2	11.7	±	11.7	1.0	±	0.6
**PDx**	65.1	±	14.5	104.4	±	41.6		*		2.8	±	2.8	9.6	±	8.1
**17-HDHA**	2789.0	±	768.0	3169.3	±	1078.3	268.5	±	183.1	177.9	±	97.9	152.3	±	52.9
**MaR1**	214.3	±	7.3	281.3	±	109.6	137.0	±	113.0	33.8	±	33.8	22.1	±	7.9
**MaR2**	21.0	±	3.2	26.4	±	8.0	6.0	±	6.0		*		0.6	±	0.6

‡Quantification of lipid mediators were as reported in Table S2 and in Methods. *denotes lipid mediator that does not meet the criteria for identification. These criteria are: MS/MS library fit score >70 with a signal-to-noise ratio > 5 (see Methods). See Fig. 3b for statistical significance among different time groups.

Table S4. Leukocyte composition of peritoneal exudates: Zymosan-induced peritonitis with low-dose SPMs

	PMN numbers [%]	Monocyte numbers [%]	Macrophage numbers [%]
12h			
**Zymosan (6)**	9.66±0.55 x 10^6 [62.84±2.05%]	0.84±0.11x 10^6 [6.30±1.00%]	1.40±0.06x 10^6 [9.07±0.52]
**Zymosan + 1X SPMs (4)**	10.02±0.40 x 10^6 [69.36±2.29%]	0.54±0.18 x 10^6 [3.82±0.89%]	1.02±0.09x 10^6 [7.07±0.57]
**Zymosan + 6X SPMs (4)**	5.91±0.54 x $10^{6\ ***}$ [67.33±2.37%]	0.36±0.46 x 10^6 [4.20±0.47%]	0.72±0.06x 10^{6***} [8.25±0.54]
24h			
**Zymosan (3)**	5.32±1.02 x 10^6 [51.65±5.55%]	0.26±0.05 x 10^6 [2.58±0.30%]	2.60±0.22x 10^6 [25.94±1.07%]
**Zymosan + 1X SPMs (4)**	5.96±0.59 x 10^6 [54.04±3.52%]	0.36±0.02 x 10^6 [3.28±0.26%]	2.73±0.45x 10^6 [24.66±3.57%]
**Zymosan + 6X SPMs (4)**	1.82±0.81 x $10^{6\ *}$ [33.91±16.41%]	0.25±0.03 x 10^6 [5.18±0.72%]$^{\#}$	1.82±0.75x 10^6 [34.19±10.08%]

Cell numbers and percentages are expressed as mean±SEM, calculated using GraphPad Prism (Column analyses - Descriptive statistics). Mouse numbers for each group are denoted within the brackets on the first column. ***$P<0.001$ vs Zymosan 12h, *$P<0.05$, $^{\#}P<0.05$ vs Zymosan 24h, using one-way ANOVA with Tukey's multiple comparison test. Also see Fig. S7 for additional statistical significance among treatment groups.

Table S5. Transcripts of SPM biosynthetic enzymes, SPM and eicosanoid receptors identified in inflammatory peritoneal exudates using RNA-seq: Log_2FC (fold change) by repetitive SPM dosing‡

Gene.name	log_2FC	P value
Alox5ap	-0.11	0.57
Alox15	0.04	0.89
Alox5	0.13	0.57
Ptgs1	0.10	0.68
Ptgs2	0.29	0.20
Lta4h	-0.14	0.09
Ltc4s	-0.24	0.49
Mgst3	-0.68	0.03
Fpr2	-0.01	0.95
Gpr18	0.35	0.21
Cmklr1	-0.14	0.50
Ltb4r1	0.09	0.68

‡ Abbreviations and nomenclature

Gene name	Receptor	Ligand	Nomenclature
**Fpr2**	ALX/FPR2	LXA_4, RvD1	
**Gpr18**	GPR18	RvD2	DRV2
**Cmklr1**	ChemR23	RvE1, RvE2	ERV1
**Ltb4r1**	BLT1	LTB_4, RvE1	

Gene name	Enzyme name
**Alox5ap**	Arachidonate 5-Lipoxygenase Activating Protein
**Alox15**	Arachidonate 12/15-Lipoxygenase
**Alox5**	Arachidonate 5-Lipoxygenase
**Ptgs1**	Prostaglandin-Endoperoxide Synthase 1
**Ptgs2**	Prostaglandin-Endoperoxide Synthase 2
**Lta4h**	Leukotriene A4 Hydrolase
**Ltc4s**	Leukotriene C4 Synthase
**Mgst3**	Microsomal Glutathione S-Transferase 3

Table S6. Gene ontology pathways that are significantly regulated by SPMs and the genes in each pathway

Genes	GO Process_name	Adj P-value	enrichment effect
Angpt1; Cth; Cyr61; Gas1; Grem1; Pdgfrb; Twist1;	negative regulation of apoptotic process	0.046530011	8.48041463
Cx3cl1; Cyr61; Itgb4; Ptprk; Sdk1; Thbs2; Tnc; Wisp2;	cell adhesion	0.021498266	9.21283502
Clnk; Cx3cl1; Cxcl13; Igkv8-16; Prg4; Tnfsf11;	immune response	0.025547854	11.838643
Angpt1; Cx3cl1; Pdgfc; Pdgfrb;	positive regulation of ERK1 and ERK2 cascade	0.051419505	12.8199768
Angpt1; Ccbe1; Ereg; Grem1; Mmp2;	angiogenesis	0.032024534	13.4609756
Ddr2; Ereg; Fgf7; Gas1; Gdnf; Grem1; Il11; Pdgfc; Pdgfrb; S100b; Tnc	positive regulation of cell proliferation	0.00057398	14.0573479
Bdkrb1; Mmp2; Pdgfrb; Ptprk; Sdc2;	cell migration	0.025804737	14.3541209
Bdkrb1; Cth; Gas1; Grem1;	negative regulation of cell growth	0.025601737	20.0245918
Cx3cl1; Cxcl13; Cyr61; Pdgfrb;	chemotaxis	0.025601737	20.3611396
Angpt1; Bdkrb1; Kirrel;	negative regulation of protein phosphorylation	0.051419505	20.4183338
Angpt1; Igfbp5; Tnfsf11;	positive regulation of protein kinase B (Akt) signaling	0.051419505	21.3791966
Cxcl13; Cyr61; Ereg; Grem1; Wisp2;	cell-cell signaling	0.009553628	22.4349593
Angpt1; Fgf7; Gdnf; Il11;	positive regulation of peptidyl-tyrosine phosphorylation	0.020557339	22.8582605
Cx3cl1; Ereg; Pdgfrb;	positive regulation of smooth muscle cell proliferation	0.048913545	23.6004118
Fgf7; Gas1; Twist1;	positive regulation of epithelial cell proliferation	0.047701015	24.2297561
Ism1; Serpinf1; Thbs2;	negative regulation of angiogenesis	0.046530011	24.893585
Cemip; Cx3cl1; Cyr61; Foxf1; Mmp2; Mmp3; Pdgfc; Pdgfrb;	positive regulation of cell migration	0.000477819	25.5050064
Angpt1; Pdgfc; Pdgfrb;	positive regulation of phosphatidylinositol 3-kinase signaling	0.034184758	28.3942454
Cyr61; Ddr2; Ereg;	positive regulation of protein kinase activity	0.032359452	29.7906837
Angpt1; Clnk; Ddr2; Gdnf; Pdgfrb;	transmembrane receptor protein tyrosine kinase signaling	0.006117214	30.2871951
Antxr1; Dlx3; Foxf1; Pdgfrb;	blood vessel development	0.009553628	34.126417
Pdgfc; Pdgfrb; Tnfsf11;	positive regulation of MAP kinase activity	0.025547854	35.6319943
Ddr2; Ereg; Pdgfc; Pdgfrb;	positive regulation of fibroblast proliferation	0.009553628	36.7117517
Cx3cl1; Tnfsf11;	monocyte chemotaxis	0.051419505	39.0802518
Ereg; Fgf7; Pdgfc;	positive regulation of cell division	0.020557339	40.3829268
Angpt1; Mmp2; Tnc;	negative regulation of cell adhesion	0.020557339	41.3007206
Cxcl13; Tnfsf11;	lymph node development	0.051419505	41.7754415
Angpt1; Grem1;	positive regulation of receptor internalization	0.051419505	44.8699187
Cyr61; Epb41l3; Igfbp5; Wisp2;	regulation of cell growth	0.006117214	46.5956848
Cx3cl1; Gas1; Gdnf;	negative regulation of extrinsic apoptotic signaling pathway	0.013144261	55.0676275
Ereg; Mmp2;	positive regulation of innate immune response	0.050535876	55.0676275
Angpt1; Fgf7; Ptprk;	protein localization to cell surface	0.009553628	72.6892683
Cx3cl1; Lrrc32;	negative regulation of cytokine secretion	0.032359452	80.7658537
Cx3cl1; Mmp2;	negative regulation of vasoconstriction	0.020557339	134.609756
